# The association between child maltreatment and problematic alcohol use in adulthood in a large multi-ethnic cohort: the HELIUS study

**DOI:** 10.1017/S2045796022000695

**Published:** 2022-12-09

**Authors:** M. M. de Waal, A. Lok, M. van Zuiden, H. Galenkamp, A. E. Goudriaan

**Affiliations:** 1 Department of Research and Jellinek, Arkin Mental Health Care, Amsterdam, the Netherlands; 2 Amsterdam Institute for Addiction Research, Amsterdam, the Netherlands; 3 Department of Psychiatry, Amsterdam UMC, Location University of Amsterdam, Meibergdreef 9, Amsterdam, the Netherlands; 4 Amsterdam Public Health, Mental Health, Amsterdam, the Netherlands; 5 Center for Urban Mental Health, University of Amsterdam, Amsterdam, the Netherlands; 6 Amsterdam Neuroscience, Mood Anxiety Psychosis Stress and Sleep, Amsterdam, the Netherlands; 7 Department of Public and Occupation Health, Amsterdam UMC, Location University of Amsterdam, Meibergdreef 9, Amsterdam, the Netherlands; 8 Amsterdam Public Health, Health Behaviors and Chronic Diseases, Amsterdam, the Netherlands

**Keywords:** Alcohol abuse, child abuse, epidemiology, maltreatment

## Abstract

**Aims:**

There is evidence that child maltreatment is associated with problematic alcohol use later in life. However, previous epidemiological studies that have examined the link between child maltreatment and adult problematic alcohol use have not considered ethnic differences. Therefore, the purpose of the current study was to investigate the relationship between child maltreatment and adult problematic alcohol use among six ethnic groups in the Netherlands, in a large, urban sample.

**Methods:**

This study used baseline data from the Healthy Life in an Urban Setting (HELIUS) study: a large-scale, multi-ethnic prospective cohort study conducted in Amsterdam, the Netherlands. Child maltreatment, current problematic alcohol use and several potential confounders (e.g. parental alcohol use) were assessed in participants (*N* = 23 356) of Dutch, South-Asian Surinamese, African Surinamese, Ghanaian, Turkish and Moroccan origin. With logistic regression analyses, we examined effect modification by ethnicity on the association between child maltreatment and problematic alcohol use. Furthermore, we explored effect modification by ethnicity for specific types of child maltreatment, namely: physical, sexual and psychological abuse and emotional neglect.

**Results:**

Effect modification by ethnicity was present. Stronger associations between child maltreatment and problematic alcohol use were found in all ethnic minority groups compared to the Dutch reference group. Particularly strong associations between all four types of child maltreatment and alcohol use problems were found for the Moroccan origin group.

**Conclusions:**

This study adds to a growing body of evidence that child maltreatment is associated with problematic alcohol use in adulthood. In addition, our findings indicate that ethnicity impacts this relationship. Although problematic alcohol use was more prevalent in the Dutch origin group, associations with child maltreatment were stronger in ethnic minority groups. Future studies on child maltreatment and alcohol use problems should also examine ethnic disparities and should further unravel how these disparities can be explained.

## Background

Numerous studies have identified the harmful effects of adverse childhood experiences, such as child maltreatment, on adult physical and mental health and problematic substance use (Kessler *et al*., 1997; Hughes *et al*., 2017; Sunley *et al*., 2020). A review indicated that child maltreatment is associated with higher alcohol consumption and alcohol use disorders in adulthood (Keyes *et al*., 2011). The authors noted that child maltreatment is more likely to occur among children of parents with alcohol use disorders, who may engage in harmful parenting practices, and may also pass along genes increasing risk of alcohol disorders to their offspring. However, the role of heritability may be limited as several studies have indicated a persistent relationship between child maltreatment and adult risk for alcohol use disorders, after controlling for family history of alcoholism (e.g. Nelson *et al*., 2002).

Child maltreatment includes physical, sexual and psychological abuse and emotional neglect during childhood. The majority of studies on the relationship between child maltreatment and adult substance use problems have used an overall measure of child maltreatment or have focused specifically on either physical abuse or sexual abuse (e.g. Burnam *et al*., 1988; Kilpatrick *et al*., 1997; Wilsnack *et al*., 1997; Meshesha *et al*., 2019). Recent meta-analyses have demonstrated that child psychological abuse is far more prevalent compared to child sexual abuse and child physical abuse (Stoltenborgh *et al*., 2011, 2012, 2013). Only recently, several studies have indicated that child psychological abuse is associated with alcohol use problems later in life (Schwandt *et al*., 2013; Schuckher *et al*., 2018). Given the high prevalence of child maltreatment and its profound impact on adult mental health, it is of utmost importance to distinguish these different types of maltreatment and to better understand their unique impact. If we can come to a better understanding of the relationship between different forms of child maltreatment and alcohol use problems in adulthood, this has relevance for prevention and treatment of substance use disorders. In addition, it is important to consider factors that may impact these relationships, such as ethnicity.

One epidemiological study in the United States including a nationally representative sample of 34 653 Americans, indicated that severe child maltreatment was more prevalent among Native Americans and Hispanics compared to Whites (Curran *et al*., 2018). Previous research on ethnic variations in substance use disorders in the United States reported that alcohol use disorders are more prevalent in Whites and Native Americans compared to in Blacks, Hispanics and Asians (Chartier and Caetano, 2010; Wu *et al*., 2011). In the Netherlands, a recent study in the same cohort as the current study, indicated that the risk to develop alcohol use disorders was lower in most ethnic minority groups compared to those of Dutch origin, except for those of Moroccan origin, whose risk was similar to the Dutch (van Amsterdam *et al*., 2020). Unfortunately, most previous large epidemiological studies that have examined the link between child maltreatment and adult problematic alcohol use (or other substances) have not considered ethnic differences. However, some studies from the United States have indicated there may be ethnic differences with this respect. For instance, a very large study in 60 000+ participants on the relationship between childhood adversities and excessive alcohol use indicated that the relationship between child abuse and heavy drinking was stronger in non-Hispanic Blacks compared to non-Hispanic Whites (Lee and Chen, 2017). Furthermore, the relationship between a broad set of household challenges (e.g. household drug abuse, parental divorce, parental intimate partner violence) and heavy drinking was stronger in non-Hispanic blacks and Hispanics compared to non-Hispanic whites. Sex did not moderate the relationship between childhood adversities and excessive alcohol use. In a longitudinal twin study in women, genes by environment effects were present in European American women, but not in African American women (Sartor *et al*., 2018). Specifically, in European American women, genetic influences on alcohol use measures were less prominent when child maltreatment had been experienced. Data on cannabis initiation and cannabis use from the same study found that child maltreatment was related to cannabis initiation and cannabis problems in both European American and African American women, but that type of environmental influences and sources of covariation between maltreatment and cannabis differed by ethnicity (Grant *et al*., 2017). In a study in adolescent/young adult men on racial differences in associations between child maltreatment and heavy drinking and other measures, no significant race-by-maltreatment effect on heavy alcohol use was found (Lee *et al*., 2012). The authors suggested this may be due to limited statistical power given the relatively small number of maltreated youth in their study. In another study including an ethnically diverse sample of college students, moderate child maltreatment was related to tobacco exposure, but not excess alcohol use (Krinner *et al*., 2020). Black respondents had significantly lower odds for both substance outcomes, but unfortunately the authors did not study ethnicity-by-maltreatment interactions on substance outcomes. Taken together, there is some evidence that the association between child maltreatment and adult alcohol use problems differs across ethnic groups. However, all previous studies focused on ethnic differences in the United States and most studies were conducted in specific subgroups. Research in other than American ethnic subgroups is lacking and it is unclear whether the association between child maltreatment and alcohol use is affected by ethnicity in a European context. Multi-ethnic comparisons of the link between child maltreatment and adult alcohol use problems in ethnic subgroups in Europe are necessary to better understand the relationship between child maltreatment and problematic alcohol use, and ultimately to improve screening, prevention and treatment of child maltreatment and alcohol use problems.

Therefore, the purpose of the current study was to investigate the relationship between child maltreatment and (current) problematic alcohol use among six ethnic groups (Dutch, South-Asian Surinamese, African Surinamese, Ghanaian, Turkish and Moroccan) in the Netherlands in a large, urban sample. Our first aim was to examine effect modification by ethnicity on the association between any child maltreatment and problematic alcohol use. We hypothesised that this association would be stronger in all non-Dutch ethnicities compared to the Dutch reference group, in line with the study by Lee and Chen (2017) in the United States, reporting a stronger association in Blacks compared to Whites. Our second aim was to examine effect modification by ethnicity on the associations between specific types of child maltreatment (emotional neglect, psychological abuse, physical abuse, sexual abuse) and problematic alcohol use. We hypothesised that all four associations would be stronger in all non-Dutch ethnicities compared to the Dutch reference group. Our final aim was to explore the independent contribution of each type of child maltreatment as predictor of problematic alcohol use in each ethnic group separately. Although causality cannot be inferred from this study, we adjusted all associations by age, sex, education level and parental alcohol use to minimise potential confounding by these demographic factors and heritability.

## Methods

### Participants and procedure

This study used baseline data from the Healthy Life in an Urban Setting (HELIUS) study: a large-scale, multi-ethnic prospective cohort study conducted in Amsterdam, the Netherlands (Stronks *et al*., 2013; Snijder *et al*., 2017). The aim of the HELIUS study is to increase knowledge and understanding of ethnic differences in major physical and mental health disorders (Stronks *et al*., 2013). As described in more detail previously (Stronks *et al*., 2013; Snijder *et al*., 2017), participants aged between 18 and 70 years were randomly sampled, stratified by ethnicity, based on information from the Amsterdam municipality register. Potential participants of Dutch, Surinamese, Turkish, Moroccan and Ghanaian backgrounds were posted an invitation to participate in the study, alongside study information and a response card. Non-Dutch persons who did not respond to the written invitation letter were visited at home by an ethnically matched interviewer to provide additional information if needed (e.g. due to language or literacy problems). Contact could be made with 55% of those invited and from those who were contactable, approximately 50% agreed to participate, which resulted in a total response rate of 28% (33% from Dutch, 31% from Surinamese, 22% from Turkish, 21% from Moroccans and 35% from Ghanaians). Written informed consent was obtained from respondents. Questionnaires were available in Dutch, English and Turkish. Participants who were unable to complete the questionnaire themselves were offered assistance from a trained ethnically matched interviewer, speaking their preferred language. The HELIUS study received approval by the Institutional Review Board of the Amsterdam UMC at the University of Amsterdam.

Baseline data collection took place from January 2011 to December 2015. From the total sample of participants who filled in the HELIUS questionnaire (*N* = 23 942), the current study excluded those of Javanese Surinamese origin (*n* = 250) and other/unknown Surinamese origin (*n* = 286) due to their comparatively small numbers. Furthermore, those of unknown ethnic background were excluded (*n* = 50). This resulted in a total study sample of 23 356 participants.

### Measures

#### Problematic alcohol use

Problematic alcohol use was measured with the Alcohol Use Disorder Identification Test (AUDIT; Saunders *et al*., 1993), a 10-item self-report questionnaire developed for screening for alcohol use disorders, with a sum score ranging from 0 to 40. Problematic alcohol use was operationalised as a dichotomous variable, with a cut-off score of ⩾8 indicating the presence of problematic alcohol use (Saunders *et al*., 1993; Babor *et al*., 2001).

Validity studies have found very favourable sensitivity and acceptable specificity for the originally recommended and commonly used cut-off score of 8 (Allen *et al*., 1997; Babor *et al*., 2001) and this cut-off score allows for comparability to other studies. However, several more recent studies have indicated that the cut-off score of 8 is suitable for males but too high for females, and have suggested differential cut-off scores for females of 5 (Aalto *et al*., 2009) or 6 (e.g. Bergman and Kallmen, 2002; Aalto *et al*., 2006; Ballester *et al*., 2021), since women develop alcohol-related problems at lower levels of consumption than men (e.g. Bradley *et al*., 1998). Therefore, additional analyses will be conducted with a cut-off score of ⩾8 for males and ⩾6 for females and presented in the online Supplement.

#### Child maltreatment

Child maltreatment was measured using a self-reported version of the 4-item NEMESIS childhood trauma scale (de Graaf *et al*., 2004). For child maltreatment type, participants were asked to indicate whether they had experienced that type ‘never’, ‘once’, ‘sometimes’ or ‘regularly’. The presence of the specific types of child maltreatment were defined as dichotomous variables (yes/no), considered ‘yes’ when the participant had indicated that they had experienced emotional neglect, psychological abuse or physical abuse ‘sometimes’ or ‘regularly’ or sexual abuse ‘once’, ‘sometimes’ or ‘regularly’, before their 16th birthday. An explanation and examples of each type of child maltreatment were provided before each relevant question. The variable ‘any child maltreatment’ was defined as dichotomous variable (yes/no) considered ‘yes’ when at least one of the four types of child maltreatment was present. The child maltreatment questionnaire is presented in the online Supplementary material.

#### Ethnicity

Ethnicity was defined based on the participant's country of birth and the country of birth of his or her parents (Stronks *et al*., 2009). A person was defined as of non-Dutch ethnic origin if he/she fulfilled one of two criteria: (a) born outside the Netherlands with at least one parent born outside the Netherlands (first generation) or (b) born in the Netherlands with both parents born outside the Netherlands (second generation). After data collection, Surinamese subgroups (African, South-Asian, Javanese, other) were determined using participant's self-reported ethnicity from the questionnaire. For the Dutch sample, people who were born in the Netherlands and whose parents were both born in the Netherlands were invited.

#### Covariates

Age and sex were derived from the municipal registry. The covariates education level and parental alcohol misuse were administered by questionnaire. Education level was categorised into four levels: low (none or elementary schooling only), low-medium (lower vocational or lower secondary schooling), medium-high (intermediate vocational or intermediate/higher secondary schooling), and high (higher vocational schooling or university). Parental alcohol misuse was measured by the following question from the NEMESIS-survey: ‘Has one or both of your parents ever abused alcohol? By “alcohol abuse” we mean a period when using alcohol caused problems.’

### Statistical analyses

Data were analysed using R version 3.4.4. There were 3968 participants (17.0%) with missing values, on either problematic alcohol use, the child maltreatment questionnaire and/or one of the covariates education level or parental alcohol misuse. The number of missing values per variable is specified in the footnote of Table 1. Missingness was significantly related to older age, female sex, lower education level, less alcohol use problems, more child maltreatment and non-Dutch ethnicity. In participants with Dutch ethnicity, 9.8% had at least one missing value, whereas in other ethnicities this varied between 16.5% in Asian Surinamese participants and 22.2% in Ghanaian participants. Missing values were assumed to be missing-not-at-random and were therefore imputed using multiple imputation by chained equations. We created 10 multiple imputed datasets with 30 iterations, using the R package mice version 2.46.0 (van Buuren and Groothuis-Oudshoorn, 2011). Predictive mean matching was used to account for the non-normal distribution of the data. All variables included in the regression analyses and types of missingness (blank *v*. rather would not say) were included in the imputation model as auxiliary variables. All results were based on the multiple imputed datasets and were pooled using Rubin's rules (Rubin, 1987).

**Table 1.** Characteristics of the study population (unimputed)

Characteristic	Total sample (*N* = 23 356)	Dutch (*n* = 4641)	South-Asian Surinamese (*n* = 3369)	African Surinamese (*n* = 4458)	Ghanaian (*n* = 2484)	Turkish (*n* = 4067)	Moroccan (*n* = 4337)
Age, years, mean (s.d.)	43.79 (13.39)	46.14 (14.05)	45.11 (13.53)	47.58 (12.77)	44.24 (11.51)	39.90 (12.44)	39.73 (13.05)
Female sex, %	57.5	54.1	53.7	59.5	61.4	54.9	62.2
Education level^a^,^b^, %							
1	17.8	3.3	14.1	5.7	28.0	31.3	30.4
2	26.6	14.3	33.4	36.2	40.0	25.0	18.2
3	29.7	22.1	29.9	35.8	25.8	29.1	34.2
4	25.9	60.3	22.5	22.3	6.2	14.5	17.2
AUDIT sum score^c^, mean (s.d.)	2.44 (4.01)	5.67 (4.55)	2.43 (4.16)	2.52 (3.53)	1.79 (3.37)	1.10 (3.06)	0.51 (2.46)
AUDIT sum score^c^ ⩾ 8, %	9.4	25.3	8.4	6.9	6.1	4.1	2.4
AUDIT sum score^c^ ⩾ 8 for males; ⩾6 for females, %	11.6	32.8	9.4	8.5	7.4	4.5	2.6
Any child maltreatment^d^, %	30.4	36.0	33.4	36.2	24.6	28.3	21.5
Types of child maltreatment, %							
Emotional neglect^e^	23.1	27.9	25.2	24.4	15.4	25.7	16.5
Psychological abuse^f^	15.0	15.8	18.9	19.1	10.3	12.5	12.0
Physical abuse^g^	14.8	9.4	19.1	22.2	16.0	11.7	12.0
Sexual abuse^h^	7.9	11.6	7.2	13.6	6.4	2.7	4.3
Parental alcohol misuse^i^, %	9.3	11.8	21.4	11.2	5.1	4.4	1.9

AUDIT, alcohol use disorder identification test; s.d., standard deviation

a (1) no education or elementary education only; (2) lower vocational or general secondary education; (3) intermediate vocational or higher secondary education; (4) higher vocational education or university.

b Missing for 208 participants.

c Missing for 208 participants.

d Missing for 1321 participants.

e Missing for 1274 participants.

f Missing for 1165 participants.

g Missing for 1005 participants.

h Missing for 944 participants.

i Missing for 2196 participants.

First, a logistic regression analysis was performed in the total sample to examine the association between the independent variable any child maltreatment and the dependent variable problematic alcohol use, adjusted for confounding by age, sex, education level, parental alcohol misuse and ethnicity. The *E*-value (vanderWeele and Ding, 2017) was calculated to understand the impact of unmeasured confounders on the observed association.

Subsequently, a logistic regression analysis was performed in the total sample to examine effect modification by ethnicity on the association between the independent variable any child maltreatment and the dependent variable problematic alcohol use, adjusted for confounding by age, sex, education level and parental alcohol use, by adding ethnicity and an interaction term (child maltreatment × ethnicity) to the analysis with Dutch ethnicity as the reference group. If effect modification by ethnicity was present (*p* < 0.05), we performed six logistic regression analyses to further explore the association between independent variable any child maltreatment and dependent variable problematic alcohol use in each ethnic group, adjusted for confounding by age, sex education level and parental alcohol use.

Subsequently, four logistic regression analyses were performed in the total sample to examine effect modification by ethnicity on the associations between the independent variables (1) emotional neglect, (2) psychological abuse, (3) physical abuse and (4) sexual abuse and the dependent variable problematic alcohol use, adjusted for confounding by age, sex, education level and parental alcohol use, with Dutch ethnicity as the reference group. If effect modification by ethnicity was present (*p* < 0.05) we performed logistic regression analyses stratified by ethnicity to further explore these four associations in each ethnic group, adjusted for confounding by age, sex, education level and parental alcohol use.

Subsequently, if effect modification was present between the child maltreatment types and problematic alcohol use, for each ethnic group we conducted a logistic regression analysis with problematic alcohol use as dependent variable, including all four types of child maltreatment as independent variables, adjusted for confounding by age, sex and education level and parental alcohol use, to explore the independent contribution of each type of child maltreatment as predictor of problematic alcohol use in each ethnic group.

Finally all logistic regression analyses were repeated with a different cut-off score for the dependent variable problematic alcohol use for females (AUDIT sum score ⩾ 8 for males, AUDIT sum score ⩾ 6 for females) to test the robustness of our findings. A significance level of *α* = 0.05 was used for all analyses.

## Results

### Sample characteristics

Participant characteristics are presented in Table 1. The majority of the sample was female (57.5%) and participants were on average 43.8 years old (s.d. = 13.4). Of the total sample, 30.4% reported any form of child maltreatment, ranging from 21.5% in Moroccans to 36.2% in African Surinamese. Problematic alcohol use (AUDIT sum score ⩾ 8) was reported by 9.4% of the total sample, ranging from 2.4% in Moroccans to 25.3% in the Dutch.

### Association between any child maltreatment and problematic alcohol use

As shown in online Supplementary Table S1, a statistically significant association was found between any child maltreatment and problematic alcohol use, adjusted for confounding by age, sex, education level, parental alcohol misuse and ethnicity. The following confounders were associated with problematic alcohol use: younger age, female sex, parental alcohol misuse and all non-Dutch ethnicities. Medium-high education level was negatively associated with problematic alcohol use, compared to the reference group low education level.

The *E*-value (vanderWeele and Ding, 2017) was 2.62 for the estimate and 2.28 for the confidence interval. Thus, an unmeasured confounder that was associated with both any child maltreatment and alcohol use problems by odds ratios of 2.62-fold each, above and beyond the measured confounders, could explain away the estimate, but weaker confounding could not. An unmeasured confounder that was associated with both any child maltreatment and alcohol use problems by odds ratios of 2.28-fold each, above and beyond the measured confounders, could explain away the lower confidence limit, but weaker confounding could not. The evidence for causality from the *E*-value thus looks reasonably strong as it would take substantial unmeasured confounding to reduce the observed association to null.

### Effect modification by ethnicity on the associations between any child maltreatment and problematic alcohol use

Effect modification by ethnicity on the association between any child maltreatment and problematic alcohol use was present. As shown in Table 2, interaction terms were significant for all non-Dutch ethnicities, indicating stronger associations between any child maltreatment and problematic alcohol use in all non-Dutch ethnicities compared to the Dutch reference group. The full model including all covariates is presented in online Supplementary Table S2. As shown in Table 3, a statistically significant association between any child maltreatment and problematic alcohol use was found in all ethnic groups. Full models per ethnicity are presented in online Supplementary Table S3.

**Table 2.** Effect modification of ethnicity on the association between (any) child maltreatment and problematic alcohol use (AUDIT ⩾ 8), with Dutch ethnicity as reference group

		95% CI for OR		
	OR	LL	UL	*p*
Any child maltreatment	1.21	1.04	1.40	0.013
Ethnicity: South-Asian Surinamese	0.19	0.16	0.23	<0.001
Ethnicity: African Surinamese	0.20	0.17	0.24	<0.001
Ethnicity: Ghanaian	0.16	0.12	0.20	<0.001
Ethnicity: Turkish	0.08	0.06	0.10	<0.001
Ethnicity: Moroccan	0.05	0.03	0.06	<0.001
Any child maltreatment × South-Asian Surinamese	1.56	1.16	2.09	0.003
Any child maltreatment × African Surinamese	1.38	1.04	1.83	0.026
Any child maltreatment × Ghanaian	1.86	1.28	2.72	0.001
Any child maltreatment × Turkish	1.92	1.35	2.72	<0.001
Any child maltreatment × Moroccan	2.81	1.84	4.31	<0.001

AUDIT, alcohol use disorder identification test; CI, confidence interval; LL, lower level; OR, odds ratio; UL, upper level

Associations are adjusted for age, sex, education level and parental alcohol misuse.

Pooled results based on multiple imputed datasets.

Full model including all covariates is presented in online Supplementary Table S2.

**Table 3.** Association between (any) child maltreatment and problematic alcohol use (AUDIT ⩾ 8) per ethnicity

		95% CI for OR		
	OR	LL	UL	*p*
Dutch	1.26	1.08	1.46	0.003
South-Asian Surinamese	1.97	1.50	2.58	<0.001
African Surinamese	1.71	1.33	2.19	<0.001
Ghanaian	2.27	1.60	3.23	<0.001
Turkish	2.19	1.57	3.07	<0.001
Moroccan	3.20	2.13	4.82	<0.001

AUDIT, alcohol use disorder identification test; CI, confidence interval; LL, lower level; OR, odds ratio; UL, upper level

Associations are adjusted for age, sex, education level and parental alcohol misuse.

Pooled results based on multiple imputed datasets.

Full models including all covariates are presented in online Supplementary Table S3.

### Effect modification by ethnicity on the associations between specific types of child maltreatment and problematic alcohol use

Table 4 shows the results of the four logistic regression analyses to examine effect modification by ethnicity on the associations between the specific types of child maltreatment and problematic alcohol use. Full models including all covariates are presented in online Supplementary Table S4. In general, the association between specific child maltreatment types and problematic alcohol use was stronger in the ethnic minority groups compared to the Dutch.

**Table 4.** Effect modification of ethnicity on the associations between specific types of child maltreatment and problematic alcohol use (AUDIT ⩾ 8), with Dutch ethnicity as reference group

		95% CI for OR		
	OR	LL	UL	*p*
Child maltreatment type: emotional neglect				
Emotional neglect	1.23	1.05	1.45	0.010
Ethnicity: South-Asian Surinamese	0.20	0.17	0.25	<0.001
Ethnicity: African Surinamese	0.21	0.17	0.25	<0.001
Ethnicity: Ghanaian	0.18	0.15	0.23	<0.001
Ethnicity: Turkish	0.09	0.07	0.11	<0.001
Ethnicity: Moroccan	0.05	0.04	0.07	<0.001
Emotional neglect × South-Asian Surinamese	1.41	1.03	1.93	0.031
Emotional neglect × African Surinamese	1.40	1.03	1.90	0.032
Emotional neglect × Ghanaian	1.47	0.94	2.29	0.092
Emotional neglect × Turkish	1.53	1.05	2.22	0.026
Emotional neglect × Moroccan	2.54	1.63	3.98	<0.001
Child maltreatment type: psychological abuse				
Psychological abuse	0.98	0.80	1.19	0.817
Ethnicity: South-Asian Surinamese	0.20	0.17	0.24	<0.001
Ethnicity: African Surinamese	0.20	0.17	0.24	<0.001
Ethnicity: Ghanaian	0.18	0.15	0.23	<0.001
Ethnicity: Turkish	0.09	0.07	0.11	<0.001
Ethnicity: Moroccan	0.05	0.04	0.07	<0.001
Psychological abuse × South-Asian Surinamese	1.65	1.16	2.34	0.005
Psychological abuse × African Surinamese	1.78	1.26	2.51	0.001
Psychological abuse × Ghanaian	1.61	0.96	2.70	0.072
Psychological abuse × Turkish	2.18	1.40	3.40	0.001
Psychological abuse × Moroccan	3.36	2.07	5.45	<0.001
Child maltreatment type: physical abuse				
Physical abuse	1.07	0.84	1.37	0.561
Ethnicity: South-Asian Surinamese	0.20	0.16	0.23	<0.001
Ethnicity: African Surinamese	0.21	0.17	0.24	<0.001
Ethnicity: Ghanaian	0.16	0.13	0.20	<0.001
Ethnicity: Turkish	0.08	0.07	0.11	<0.001
Ethnicity: Moroccan	0.04	0.03	0.06	<0.001
Physical abuse × South-Asian Surinamese	1.76	1.22	2.56	0.003
Physical abuse × African Surinamese	1.44	1.01	2.06	0.044
Physical abuse × Ghanaian	2.11	1.34	3.33	0.001
Physical abuse × Turkish	2.52	1.61	3.93	<0.001
Physical abuse × Moroccan	4.60	2.85	7.45	<0.001
Child maltreatment type: sexual abuse				
Sexual abuse	1.46	1.16	1.83	0.001
Ethnicity: South-Asian Surinamese	0.22	0.19	0.26	<0.001
Ethnicity: African Surinamese	0.23	0.20	0.27	<0.001
Ethnicity: Ghanaian	0.19	0.16	0.24	<0.001
Ethnicity: Turkish	0.10	0.08	0.12	<0.001
Ethnicity: Moroccan	0.06	0.05	0.07	<0.001
Sexual abuse × South-Asian Surinamese	1.41	0.85	2.33	0.186
Sexual abuse × African Surinamese	0.93	0.60	1.45	0.756
Sexual abuse × Ghanaian	1.11	0.58	2.12	0.751
Sexual abuse × Turkish	1.94	0.92	4.09	0.083
Sexual abuse × Moroccan	3.57	1.97	6.46	<0.001

AUDIT, alcohol use disorder identification test; CI, confidence interval; LL, lower level; OR, odds ratio; UL, upper level

Associations are adjusted for age, sex, education level and parental alcohol misuse.

Pooled results based on multiple imputed datasets.

Full models including all covariates are presented in online Supplementary Table S4.

For emotional neglect and for psychological abuse, statistically significant effect modification was present for the South-Asian Surinamese, African Surinamese, Turkish and Moroccan ethnicities, indicating stronger associations between these types of child maltreatment and problematic alcohol use in these ethnicities compared to the Dutch reference group. For physical abuse, statistically significant effect modification was present for all non-Dutch ethnicities, indicating stronger associations between physical abuse and problematic alcohol use in all non-Dutch ethnicities compared to the Dutch reference group. For sexual abuse, statistically significant effect modification was present for the Moroccan ethnicity, indicating a stronger association between sexual abuse and problematic alcohol use for the Moroccan ethnicity compared to the Dutch reference group. Since effect modification was present, Table 5 shows the results of the explorative regression analyses between the four child maltreatment types and problematic alcohol use stratified by ethnicity. Full models including all covariates are presented in online Supplementary Table S5. The results indicate that physical abuse is significantly associated with problematic alcohol use in all ethnicities, except for the Dutch. In addition, particularly high ORs were found for the associations between all four types of child maltreatment and alcohol use problems for the Moroccan ethnicity.

**Table 5.** Associations between each type of child maltreatment and problematic alcohol use (AUDIT ⩾ 8) stratified by ethnicity

		95% CI for OR		
	OR	LL	UL	*p*
Emotional neglect				
Dutch	1.31	1.12	1.55	0.001
South-Asian Surinamese	1.78	1.33	2.39	<0.001
African Surinamese	1.75	1.34	2.29	<0.001
Ghanaian	1.77	1.15	2.70	0.009
Turkish	1.77	1.23	2.54	0.002
Moroccan	3.05	1.97	4.73	<0.001
Psychological abuse				
Dutch	1.05	0.86	1.28	0.654
South-Asian Surinamese	1.67	1.22	2.29	0.002
African Surinamese	1.71	1.28	2.29	<0.001
Ghanaian	1.52	0.93	2.50	0.099
Turkish	1.97	1.28	3.01	0.002
Moroccan	3.22	2.03	5.11	<0.001
Physical abuse				
Dutch	1.16	0.91	1.47	0.228
South-Asian Surinamese	1.92	1.43	2.59	<0.001
African Surinamese	1.53	1.17	2.01	0.002
Ghanaian	2.24	1.52	3.31	<0.001
Turkish	2.41	1.62	3.58	<0.001
Moroccan	4.64	3.03	7.12	<0.001
Sexual abuse				
Dutch	1.39	1.11	1.75	0.004
South-Asian Surinamese	2.75	1.65	4.56	<0.001
African Surinamese	1.44	0.96	2.16	0.082
Ghanaian	1.71	0.92	3.19	0.092
Turkish	3.16	1.46	6.84	0.004
Moroccan	5.02	2.78	9.07	<0.001

AUDIT, alcohol use disorder identification test; CI, confidence interval; LL, lower level; OR, odds ratio; UL, upper level

Associations are adjusted for age, sex, education level and parental alcohol misuse.

Pooled results based on multiple imputed datasets.

Full models including all covariates are presented in online Supplementary Table S5a–S5d.

### Exploring the independent contribution of types of child maltreatment as predictors of problematic alcohol use for each ethnic group

Table 6 shows the results of the logistic regression analyses to explore the independent contribution of each type of child maltreatment as predictor of problematic alcohol use in each ethnic group, adjusted for confounding by age, sex, education level and parental alcohol use. The full models including all covariates are presented in online Supplementary Table S6. In general, we found that psychological abuse did not significantly independently predict alcohol use problems in any of the ethnic groups, whereas the other types of child maltreatment did in some of the groups. For the Dutch ethnicity, emotional neglect and sexual abuse were significant independent predictors of problematic alcohol use. For the South-Asian Surinamese ethnicity and Moroccan ethnicity, physical abuse and sexual abuse were significant independent predictors of problematic alcohol use. For the African Surinamese, emotional neglect was the only significant independent predictor of problematic alcohol use. For the Ghanaian and Turkish ethnicity, physical abuse was the only significant independent predictor of problematic alcohol use. A correlation matrix showing correlations (phi-coefficients) between the types of child maltreatment in the total sample and per ethnicity is presented in online Supplementary Table S7. In all ethnicities, psychological abuse was moderately correlated to both emotional neglect (*φ* = 0.61 in the total sample) and physical abuse (*φ* = 0.57 in the total sample). Therefore, psychological abuse may have had no significant independent predictive value for problematic alcohol use when all types of child maltreatment were controlled for each other.

**Table 6.** Associations between child maltreatment types and problematic alcohol use (AUDIT ⩾ 8) per ethnicity

		95% CI for OR		
	OR	LL	UL	*p*
Dutch				
Emotional neglect	1.37	1.13	1.65	0.001
Psychological abuse	0.80	0.62	1.02	0.075
Physical abuse	1.09	0.83	1.44	0.518
Sexual abuse	1.33	1.05	1.68	0.017
South-Asian Surinamese				
Emotional neglect	1.44	0.97	2.14	0.069
Psychological abuse	0.88	0.55	1.41	0.591
Physical abuse	1.56	1.05	2.31	0.027
Sexual abuse	2.12	1.25	3.59	0.005
African Surinamese				
Emotional neglect	1.46	1.00	2.14	0.049
Psychological abuse	1.20	0.76	1.89	0.436
Physical abuse	1.10	0.77	1.59	0.598
Sexual abuse	1.11	0.72	1.71	0.638
Ghanaian				
Emotional neglect	1.41	0.79	2.49	0.246
Psychological abuse	0.65	0.32	1.31	0.228
Physical abuse	2.24	1.37	3.65	0.001
Sexual abuse	1.40	0.72	2.70	0.318
Turkish				
Emotional neglect	1.25	0.79	1.99	0.345
Psychological abuse	1.08	0.61	1.92	0.781
Physical abuse	1.92	1.17	3.14	0.009
Sexual abuse	2.12	0.94	4.78	0.070
Moroccan				
Emotional neglect	1.29	0.69	2.40	0.420
Psychological abuse	1.06	0.54	2.05	0.873
Physical abuse	3.30	1.86	5.85	<0.001
Sexual abuse	2.54	1.32	4.90	0.005

AUDIT, alcohol use disorder identification test; CI, confidence interval; LL, lower level; OR, odds ratio; UL, upper level

For each ethnicity, all types of child maltreatment were included in the same model.

Associations are adjusted for age, sex, education level and parental alcohol misuse.

Pooled results based on multiple imputed datasets.

Full models including all covariates are presented in online Supplementary Table S6.

As shown in online Supplementary Table S6, for all ethnicities, male sex was significantly associated with problematic alcohol use. For the Dutch, South-Asian Surinamese, African Surinamese and Moroccan ethnicity, younger age was significantly associated with problematic alcohol use, whereas for the Ghanaian and Turkish ethnicity it was not. For all ethnicities except for the Dutch, parental alcohol misuse was significantly associated with problematic alcohol use.

### Supplementary analyses with AUDIT cut-off score of ⩾6 for females

As shown in online Supplementary Tables S8–S13, logistic regression analyses with a different cut-off score for problematic alcohol use for females (AUDIT sum score ⩾ 8 for males, AUDIT sum score ⩾ 6 for females) yielded very similar results, which supports the robustness of our findings. We did find small differences for the Ghanaian and Turkish ethnicities. For the Ghanaian ethnicity, effect modification was present for emotional neglect, and emotional neglect was a significant independent predictor of problematic alcohol use. For the Turkish ethnicity, effect modification was present for sexual abuse, and sexual abuse was a significant independent predictor of problematic alcohol use.

## Discussion

This study in a large, urban, multi-ethnic sample confirms that child maltreatment is associated with problematic alcohol use in adulthood. As hypothesised, we found that this association was stronger in all ethnic minority groups compared to the Dutch. However, effect modification by ethnicity was not consistently present for all types of child maltreatment within all minority groups. The association between physical abuse and problematic alcohol use was stronger in all ethnic minority groups compared to the Dutch. For both emotional neglect and psychological abuse, their association with problematic alcohol use was stronger in all ethnic minority groups compared to the Dutch, except for the Ghanaian origin group. In the supplementary analysis with a different AUDIT cut-off score for females, the association between emotional neglect and problematic alcohol use was also stronger in the Ghanaian origin group. The association between sexual abuse and problematic alcohol use was only significantly stronger in the Moroccan origin group compared to the Dutch; in the supplementary analysis, also in the Turkish origin group. For all types of child maltreatment, we found the largest odds ratios in the Moroccan origin group. Our findings are in line with findings by Lee and Chen (2017) reporting a stronger association between child abuse and excessive alcohol use in non-Hispanic blacks compared to non-Hispanic whites in the United States. However, another study from the United States did not find a significant ethnicity-by-maltreatment interaction effect on heavy alcohol use in adolescent/young adult men (Lee *et al*., 2012). The authors suggested this may be due to limited statistical power given the relatively small number of maltreated youth in their study. Although most previous large epidemiological studies have not considered effect-modification by ethnicity, they did examine ethnic variations in child maltreatment and substance use disorder rates. In the United States, child maltreatment has been found to be more prevalent among Native Americans and Hispanics compared to Whites (Curran *et al*., 2018); whereas substance use disorders were found to be more prevalent in Whites and Native Americans compared to in Blacks, Hispanics and Asians (Chartier and Caetano, 2010; Wu *et al*., 2011). In the current study, we found that 25.3% of the Dutch reported problematic alcohol use, whereas for the non-Dutch ethnicities this varied between 2.4 and 8.4%. This is in line with previous research in the same cohort *(*van Amsterdam *et al*., 2020). Cultural factors may contribute to problematic alcohol use being more common in the Dutch compared to ethnic minorities (van Amsterdam *et al*., 2020). This is supported by previous findings in ethnic minorities that higher acculturation is positively associated with problematic alcohol use, whereas enculturation, cultural pride and public and intrinsic religiosity are negatively associated with problematic alcohol use (Yu and Stiffman, 2007; Caetano *et al*., 2009; Amundsen, 2012; Meyers *et al*., 2017; Lui and Zamboanga, 2018). In ethnic minority groups, problematic alcohol use may be more in contrast with cultural and religious norms and therefore is less likely to arise, unless certain adversities are present, such as child maltreatment. This may explain the stronger association between child maltreatment and problematic alcohol use in ethnic minority groups compared to the Dutch.

Another explanation for our findings could be that certain additional life stressors that are more prevalent in ethnic minority groups, such as ethnic discrimination, may amplify the impact of child maltreatment on problematic substance use. A large body of evidence suggests that the association between child maltreatment and problematic substance use may be partly attributable to stress sensitivity, whereby early adversity heightens sensitivity to subsequent life stressors and increases risk for stress-related drinking or drug use (Young-Wolff *et al*., 2012; Eames *et al*., 2014; Kim *et al*., 2014; Myers *et al*., 2014; Shin *et al*., 2015). Perceived ethnic discrimination is considered an important chronic psychosocial stressor for ethnic minority groups, and has been associated with alcohol consumption (Terrell *et al*., 2006; Borrell *et al*., 2007; Gilbert and Zemore, 2016). A study in the same cohort as the current study indicated that perceived ethnic discrimination was related to alcohol outcomes in the African Surinamese and Ghanaian ethnicities (Visser *et al*., 2017).

In addition, differences in stress related to family values and obligations across ethnic groups may play a role. A Dutch study among five ethnic groups in the Netherlands found that ethnic minority groups had more traditional family values compared to the Dutch, with Moroccan and Turks having the most traditional family values, followed by Surinamese (Arends-Toth and van de Vijver, 2009). Family values were measured with statements addressing family obligations (e.g. ‘children should take care of their elderly parents’). Previous research in American adolescents from Mexican backgrounds indicated that family assistance behaviours (i.e. provision of instrumental support to the family) were associated with higher tobacco, alcohol, marijuana and illicit drug use, but only when the assistance took place within high-conflict homes (Telzer *et al*., 2014). The authors suggest that parent-child conflict may be especially distressing among families from Mexican backgrounds who tend to emphasise strong family solidarity and connection, and that adolescents who report more conflicts with their parents may experience an increased sense of burden when they assist their family, and feel greater emotional distress, which can lead to heightened substance use. Possibly, also people from families with more traditional family values and obligations who have been victims of domestic child maltreatment may feel more burdened by these obligations compared to adults from families with less traditional family values. This may be particularly true for traditional Islamic societies, in which the family is perceived as the highest social unit and the core of society, in which sustained loyalty is highly valued, and children are expected to provide shelter and adequate financial and social support for their parents when they grow into adulthood (Stepien, 2008). Victims of domestic child maltreatment from traditional families may be more at risk for drinking to cope with the burden of ongoing family obligations and sustained loyalty, compared to those from less traditional families. This might explain that we found the largest odds ratios for the associations between child maltreatment and problematic alcohol use in the Moroccan origin group. Future studies examining ethnic disparities in the relationship between child maltreatment and problematic alcohol use should incorporate the role of ethnic discrimination, cultural norms, family values and religion.

### Limitations

This study has several limitations. First, causality cannot be inferred from this study, as is the case in all child maltreatment studies. Although we adjusted for several potential confounders such as parental alcohol use, confounding by other factors, such as childhood socio-economic status and parental education cannot be ruled out. However, the evidence for causality from the *E*-value looks reasonably strong as it would take substantial unmeasured confounding to reduce the observed association to null. Although we did include education level as potential confounder, this could also be a mediator of the relationship between child maltreatment and problematic alcohol use.

Second, the response rate of study was quite low (28%) and varied between ethnic groups. Previous non-response analyses indicated that in all ethnic groups, those participating were more often women and were slightly older than those not participating (Snijder *et al*., 2017). However, these non-response analyses are limited since we have no data on non-responders other than age and sex. The lower response rates to the first written invitation in non-Dutch ethnicities might be due to language or literacy difficulties experienced. For this reason, non-Dutch ethnicities were visited at home by an ethnically matched interviewer to provide additional information if needed. Although this did increase response rates in non-Dutch ethnicities, this may have induced selection bias, since those who responded to the first written invitation letter might differ from those who did not on other factors than language problems (e.g. socio-economic status or health problems).

Third, effects of memory biases cannot be excluded since the assessment of child maltreatment was based on retrospective self-report. Although retrospective surveys of child maltreatment have been found to be sufficiently valid (Brewin *et al*., 1993), memory bias can lead to both underreporting and overreporting of actual experiences (Baldwin *et al*., 2019).

Fourth, the child maltreatment questionnaire used consists of single items for each child maltreatment type. Although it has not been validated against a more expansive measurement instrument for child maltreatment, the types of concrete maltreatment behaviours explicitly inquired upon in the questionnaire are highly similar to those inquired upon in the Childhood Trauma Interview, which was previously shown to have good convergent and divergent validity compared to other measurement instruments (Fink *et al*., 1995).

Fifth, although the child maltreatment questionnaire was developed for use in a Dutch community population also including a minority of participants from the same ethnic groups as in the current study, the questionnaire was not validated for the different ethnic groups in our study.

Sixth, although we used a dichotomous definition of child maltreatment types, the severity of child maltreatment types may be associated with problematic alcohol use in adulthood and these associations might differ across ethnic groups. Therefore, future studies should include a measure of severity of different types of child maltreatment, validated in all ethnic groups included in the study, and ideally use a prospective study design.

Seventh, since problematic alcohol use was significantly higher in the Dutch sample compared to other samples, future studies should incorporate other variables that might also explain the differences between the Dutch ethnicity and other ethnicities, such as an individual's cultural values, beliefs and norms, social life and public and intrinsic religiosity.

### Implications

The current study adds to a growing body of evidence that child maltreatment is associated with problematic alcohol use later in life. It is of utmost importance to better understand this relationship to improve prevention, screening, and treatment of both child maltreatment and alcohol use disorders. Our findings contribute to this understanding by demonstrating that ethnicity is an important factor that impacts the association between child maltreatment and problematic alcohol use in adulthood. Future studies on child maltreatment and alcohol use problems should also examine ethnic disparities and should further unravel how these disparities can be explained. It is important that families of all ethnicities have access to and come to the attention of child services, and are reached by proactive, preventive services. In addiction treatment facilities, experiences of child maltreatment and their impact should be assessed in each patient, particularly (but not exclusively) in patients from ethnic minority groups. If indicated, patients with alcohol use disorders should be offered treatment for comorbid mental health problems that may stem from child maltreatment, such as posttraumatic stress disorder (Lortye *et al*., 2021; Rameckers *et al*., 2021) or depressive disorder (Christ *et al*., 2019; Humphreys *et al*., 2020; Sunley *et al*., 2020).

## Conclusion

This study in a large multi-ethnic cohort confirms that child maltreatment is associated with problematic alcohol use in adulthood. Although problematic alcohol use was more prevalent in the Dutch origin group, associations with child maltreatment were stronger in ethnic minority groups compared to the Dutch.

## Data Availability

The HELIUS data are owned by the Amsterdam University Medical Centers, location AMC in Amsterdam, The Netherlands. Any researcher can request the data by submitting a proposal to the HELIUS Executive Board as outlined at http://www.heliusstudy.nl/en/researchers/collaboration, by email: heliuscoordinator@amsterdamumc.nl. The HELIUS Executive Board will check proposals for compatibility with the general objectives, ethical approvals and informed consent forms of the HELIUS study. There are no other restrictions to obtaining the data and all data requests will be processed in the same manner. The data cannot be shared publicly because of privacy restrictions.

## Supplementary material

For supplementary material accompanying this paper visit https://doi.org/10.1017/S2045796022000695. click here to view supplementary material
