# Supplementary material for: The association between child maltreatment and problematic alcohol use in adulthood in a large multi-ethnic cohort: the HELIUS study
Source: Epidemiol Psychiatr Sci. 2022 Dec 9;31:e87. doi: 10.1017/S2045796022000695 (PMC9762143; doi:10.1017/S2045796022000695)
Supplement: Supplementary file 1 [file S2045796022000695sup001.docx]

**Supplementary Material**

**Child maltreatment questionnaire**

The following questions are about negative experiences you may have had as a child: that is, when you were younger than 16 years of age.

1. *‘Emotional neglect’ means you feel you weren’t listened to at home, that your problems and experiences were ignored, or that you didn’t feel you could go to your parents for attention or support.*

Do you think you were emotionally neglected before your 16th birthday?

☐ Never

☐ Once

☐ Sometimes

☐ Regularly

☐ Would rather not answer

2*. ‘Psychological abuse’ is verbal abuse, unfair punishment, being disadvantaged when compared to brothers and sisters, blackmail, and so forth.*

Do you think you were psychologically (not physically) abused before your 16th birthday?

☐ Never

☐ Once

☐ Sometimes

☐ Regularly

☐ Would rather not answer

3. ‘Physical abuse’ is being kicked, hit with hands or another object, or other forms of physical abuse.

Do you think you were physically abused before your 16^th^ birthday?

☐ Never

☐ Once

☐ Sometimes

☐ Regularly

☐ Would rather not answer

4. Being ‘sexually approached’ is being touched or having to touch somebody else in a sexual way when you don’t want to.

Do you think you were sexually approached before your 16^th^ birthday?

☐ Never

☐ Once

☐ Sometimes

☐ Regularly

☐ Would rather not answer

**Supplementary Table S1**

The association between (any) child maltreatment and problematic alcohol use (AUDIT ≥ 8), adjusted for confounding by age, sex, education level, parental alcohol misuse, and ethnicity.

|  | OR | 95% CI for OR | | *P* |
| --- | --- | --- | --- | --- |
|  |  | LL | UL |  |
| Any child maltreatment | 1.62 | 1.46 | 1.79 | < .001 |
| Age | 0.98 | 0.97 | 0.98 | < .001 |
| Sex: Female | 0.23 | 0.21 | 0.26 | < .001 |
| Education level: low-medium | 0.85 | 0.71 | 1.02 | .087 |
| Education level: medium-high | 0.75 | 0.62 | 0.91 | .003 |
| Education level: high | 0.90 | 0.75 | 1.08 | .268 |
| Parental alcohol misuse | 1.67 | 1.44 | 1.94 | < .001 |
| Ethnicity: South-Asian Surinamese | 0.23 | 0.19 | 0.26 | < .001 |
| Ethnicity: African Surinamese | 0.23 | 0.19 | 0.26 | < .001 |
| Ethnicity: Ghanaian | 0.20 | 0.16 | 0.24 | < .001 |
| Ethnicity: Turkish | 0.10 | 0.08 | 0.12 | < .001 |
| Ethnicity: Moroccan | 0.07 | 0.05 | 0.08 | < .001 |

*Note.* AUDIT = alcohol use disorder identification test; CI = confidence interval; LL = lower level; OR = odds ratio; UL = upper level.

Reference group for education level = low; reference group for ethnicity = Dutch.

Pooled results based on multiple imputed datasets.

**Supplementary Table S2**

Effect modification of ethnicity on the association between (any) child maltreatment and problematic alcohol use (AUDIT ≥ 8), adjusted for confounding by age, sex, education level, parental alcohol misuse, and ethnicity.

|  | OR | 95% CI for OR | | *P* |
| --- | --- | --- | --- | --- |
|  |  | LL | UL |  |
| Any child maltreatment | 1.21 | 1.04 | 1.40 | .013 |
| Age | 0.98 | 0.97 | 0.98 | < .001 |
| Sex: Female | 0.24 | 0.21 | 0.26 | < .001 |
| Education level: low-medium | 0.85 | 0.71 | 1.02 | .088 |
| Education level: medium-high | 0.75 | 0.62 | 0.91 | .003 |
| Education level: high | 0.89 | 0.74 | 1.08 | .243 |
| Parental alcohol misuse | 1.69 | 1.46 | 1.96 | < .001 |
| Ethnicity: South-Asian Surinamese | 0.19 | 0.16 | 0.23 | < .001 |
| Ethnicity: African Surinamese | 0.20 | 0.17 | 0.24 | < .001 |
| Ethnicity: Ghanaian | 0.16 | 0.12 | 0.20 | < .001 |
| Ethnicity: Turkish | 0.08 | 0.06 | 0.10 | < .001 |
| Ethnicity: Moroccan | 0.05 | 0.03 | 0.06 | < .001 |
| Any child maltreatment * South-Asian Surinamese | 1.56 | 1.16 | 2.09 | .003 |
| Any child maltreatment * African Surinamese | 1.38 | 1.04 | 1.83 | .026 |
| Any child maltreatment * Ghanaian | 1.86 | 1.28 | 2.72 | .001 |
| Any child maltreatment * Turkish | 1.92 | 1.35 | 2.72 | < .001 |
| Any child maltreatment * Moroccan | 2.81 | 1.84 | 4.31 | < .001 |

*Note.* AUDIT = alcohol use disorder identification test; CI = confidence interval; LL = lower level; OR = odds ratio; UL = upper level.

Reference group for education level = low; reference group for ethnicity = Dutch.
Pooled results based on multiple imputed datasets.

**Supplementary Table S3**

Association between (any) child maltreatment and problematic alcohol use (AUDIT ≥ 8) per ethnicity, adjusted for confounding by age, sex, education level, and parental alcohol misuse.

|  | OR | 95% CI for OR | | *P* |
| --- | --- | --- | --- | --- |
|  |  | LL | UL |  |
| Dutch |  |  |  |  |
| Any child maltreatment | 1.26 | 1.08 | 1.46 | .003 |
| Age | 0.97 | 0.97 | 0.98 | < .001 |
| Sex: Female | 0.34 | 0.30 | 0.39 | < .001 |
| Education level: low-medium | 0.84 | 0.53 | 1.33 | .450 |
| Education level: medium-high | 1.10 | 0.71 | 1.70 | .681 |
| Education level: high | 1.24 | 0.81 | 1.90 | .312 |
| Parental alcohol misuse | 1.20 | 0.96 | 1.51 | .115 |
| South-Asian Surinamese |  |  |  |  |
| Any child maltreatment | 1.97 | 1.50 | 2.58 | < .001 |
| Age | 0.98 | 0.97 | 0.99 | < .001 |
| Sex: Female | 0.12 | 0.09 | 0.17 | < .001 |
| Education level: low-medium | 0.80 | 0.53 | 1.20 | .279 |
| Education level: medium-high | 0.56 | 0.36 | 0.87 | .010 |
| Education level: high | 0.67 | 0.43 | 1.04 | .077 |
| Parental alcohol misuse | 1.90 | 1.41 | 2.55 | < .001 |
| African Surinamese |  |  |  |  |
| Any child maltreatment | 1.71 | 1.33 | 2.19 | < .001 |
| Age | 0.98 | 0.97 | 0.99 | < .001 |
| Sex: Female | 0.21 | 0.16 | 0.27 | < .001 |
| Education level: low-medium | 0.63 | 0.41 | 0.98 | .041 |
| Education level: medium-high | 0.47 | 0.30 | 0.74 | .001 |
| Education level: high | 0.42 | 0.26 | 0.69 | .001 |
| Parental alcohol misuse | 1.99 | 1.42 | 2.79 | < .001 |
| Ghanaian |  |  |  |  |
| Any child maltreatment | 2.27 | 1.60 | 3.23 | < .001 |
| Age | 1.01 | 0.99 | 1.02 | .278 |
| Sex: Female | 0.31 | 0.21 | 0.45 | < .001 |
| Education level: low-medium | 1.09 | 0.70 | 1.70 | .698 |
| Education level: medium-high | 0.92 | 0.56 | 1.52 | .756 |
| Education level: high | 0.50 | 0.20 | 1.23 | .132 |
| Parental alcohol misuse | 2.81 | 1.59 | 4.97 | < .001 |
| Turkish |  |  |  |  |
| Any child maltreatment | 2.19 | 1.57 | 3.07 | < .001 |
| Age | 0.99 | 0.98 | 1.01 | .411 |
| Sex: Female | 0.07 | 0.04 | 0.12 | < .001 |
| Education level: low-medium | 0.80 | 0.50 | 1.28 | .358 |
| Education level: medium-high | 0.92 | 0.57 | 1.48 | .720 |
| Education level: high | 0.82 | 0.47 | 1.43 | .478 |
| Parental alcohol misuse | 3.20 | 1.86 | 5.49 | < .001 |
| Moroccan |  |  |  |  |
| Any child maltreatment | 3.20 | 2.13 | 4.82 | < .001 |
| Age | 0.97 | 0.95 | 0.99 | .006 |
| Sex: Female | 0.11 | 0.06 | 0.18 | < .001 |
| Education level: low-medium | 1.51 | 0.77 | 2.97 | .229 |
| Education level: medium-high | 1.02 | 0.52 | 2.00 | .958 |
| Education level: high | 1.64 | 0.83 | 3.26 | .158 |
| Parental alcohol misuse | 3.83 | 1.69 | 8.69 | .001 |

*Note.* AUDIT = alcohol use disorder identification test; CI = confidence interval; LL = lower level; OR = odds ratio; UL = upper level.

Reference group for education level = low.
Pooled results based on multiple imputed datasets.

**Supplementary Table S4**

Effect modification of ethnicity on the associations between specific types of child maltreatment and problematic alcohol use (AUDIT ≥ 8), adjusted for confounding by age, sex, education level, parental alcohol misuse, and ethnicity.

|  | OR | 95% CI for OR | | *P* |
| --- | --- | --- | --- | --- |
|  |  | LL | UL |  |
| Emotional neglect | 1.23 | 1.05 | 1.45 | .010 |
| Age | 0.98 | 0.97 | 0.98 | < .001 |
| Sex: Female | 0.24 | 0.22 | 0.26 | < .001 |
| Education level: low-medium | 0.86 | 0.71 | 1.04 | .111 |
| Education level: medium-high | 0.77 | 0.64 | 0.93 | .006 |
| Education level: high | 0.92 | 0.76 | 1.10 | .357 |
| Parental alcohol misuse | 1.70 | 1.47 | 1.98 | < .001 |
| Ethnicity: South-Asian Surinamese | 0.20 | 0.17 | 0.25 | < .001 |
| Ethnicity: African Surinamese | 0.21 | 0.17 | 0.25 | < .001 |
| Ethnicity: Ghanaian | 0.18 | 0.15 | 0.23 | < .001 |
| Ethnicity: Turkish | 0.09 | 0.07 | 0.11 | < .001 |
| Ethnicity: Moroccan | 0.05 | 0.04 | 0.07 | < .001 |
| Emotional neglect * South-Asian Surinamese | 1.41 | 1.03 | 1.93 | .031 |
| Emotional neglect * African Surinamese | 1.40 | 1.03 | 1.90 | .032 |
| Emotional neglect * Ghanaian | 1.47 | 0.94 | 2.29 | .092 |
| Emotional neglect * Turkish | 1.53 | 1.05 | 2.22 | .026 |
| Emotional neglect * Moroccan | 2.54 | 1.63 | 3.98 | < .001 |
| Psychological abuse | 0.98 | 0.80 | 1.19 | .817 |
| Age | 0.98 | 0.97 | 0.98 | < .001 |
| Sex: Female | 0.24 | 0.22 | 0.27 | < .001 |
| Education level: low-medium | 0.86 | 0.71 | 1.03 | .106 |
| Education level: medium-high | 0.77 | 0.64 | 0.93 | .007 |
| Education level: high | 0.92 | 0.76 | 1.11 | .393 |
| Parental alcohol misuse | 1.78 | 1.53 | 2.06 | < .001 |
| Ethnicity: South-Asian Surinamese | 0.20 | 0.17 | 0.24 | < .001 |
| Ethnicity: African Surinamese | 0.20 | 0.17 | 0.24 | < .001 |
| Ethnicity: Ghanaian | 0.18 | 0.15 | 0.23 | < .001 |
| Ethnicity: Turkish | 0.09 | 0.07 | 0.11 | < .001 |
| Ethnicity: Moroccan | 0.05 | 0.04 | 0.07 | < .001 |
| Psychological abuse * South-Asian Surinamese | 1.65 | 1.16 | 2.34 | .005 |
| Psychological abuse * African Surinamese | 1.78 | 1.26 | 2.51 | .001 |
| Psychological abuse * Ghanaian | 1.61 | 0.96 | 2.70 | .072 |
| Psychological abuse * Turkish | 2.18 | 1.40 | 3.40 | .001 |
| Psychological abuse * Moroccan | 3.36 | 2.07 | 5.45 | < .001 |
| Physical abuse | 1.07 | 0.84 | 1.37 | .561 |
| Age | 0.98 | 0.97 | 0.98 | < .001 |
| Sex: Female | 0.25 | 0.22 | 0.27 | < .001 |
| Education level: low-medium | 0.85 | 0.71 | 1.03 | .090 |
| Education level: medium-high | 0.76 | 0.63 | 0.92 | .005 |
| Education level: high | 0.90 | 0.75 | 1.09 | .284 |
| Parental alcohol misuse | 1.76 | 1.52 | 2.03 | < .001 |
| Ethnicity: South-Asian Surinamese | 0.20 | 0.16 | 0.23 | < .001 |
| Ethnicity: African Surinamese | 0.21 | 0.17 | 0.24 | < .001 |
| Ethnicity: Ghanaian | 0.16 | 0.13 | 0.20 | < .001 |
| Ethnicity: Turkish | 0.08 | 0.07 | 0.11 | < .001 |
| Ethnicity: Moroccan | 0.04 | 0.03 | 0.06 | < .001 |
| Physical abuse * South-Asian Surinamese | 1.76 | 1.22 | 2.56 | .003 |
| Physical abuse * African Surinamese | 1.44 | 1.01 | 2.06 | .044 |
| Physical abuse * Ghanaian | 2.11 | 1.34 | 3.33 | .001 |
| Physical abuse * Turkish | 2.52 | 1.61 | 3.93 | < .001 |
| Physical abuse * Moroccan | 4.60 | 2.85 | 7.45 | < .001 |
| Sexual abuse | 1.46 | 1.16 | 1.83 | .001 |
| Age | 0.98 | 0.97 | 0.98 | < .001 |
| Sex: Female | 0.23 | 0.21 | 0.26 | < .001 |
| Education level: low-medium | 0.84 | 0.70 | 1.01 | .068 |
| Education level: medium-high | 0.75 | 0.62 | 0.91 | .003 |
| Education level: high | 0.90 | 0.75 | 1.09 | .288 |
| Parental alcohol misuse | 1.82 | 1.57 | 2.11 | < .001 |
| Ethnicity: South-Asian Surinamese | 0.22 | 0.19 | 0.26 | < .001 |
| Ethnicity: African Surinamese | 0.23 | 0.20 | 0.27 | < .001 |
| Ethnicity: Ghanaian | 0.19 | 0.16 | 0.24 | < .001 |
| Ethnicity: Turkish | 0.10 | 0.08 | 0.12 | < .001 |
| Ethnicity: Moroccan | 0.06 | 0.05 | 0.07 | < .001 |
| Sexual abuse * South-Asian Surinamese | 1.41 | 0.85 | 2.33 | .186 |
| Sexual abuse * African Surinamese | 0.93 | 0.60 | 1.45 | .756 |
| Sexual abuse * Ghanaian | 1.11 | 0.58 | 2.12 | .751 |
| Sexual abuse * Turkish | 1.94 | 0.92 | 4.09 | .083 |
| Sexual abuse * Moroccan | 3.57 | 1.97 | 6.46 | < .001 |

*Note.* AUDIT = alcohol use disorder identification test; CI = confidence interval; LL = lower level; OR = odds ratio; UL = upper level.

Reference group for education level = low; reference group for ethnicity = Dutch.
Pooled results based on multiple imputed datasets.

**Supplementary Table S5a**

Association between emotional neglect and problematic alcohol use (AUDIT ≥ 8) per ethnicity, adjusted for confounding by age, sex, education level, and parental alcohol misuse.

|  | OR | 95% CI for OR | | *P* |
| --- | --- | --- | --- | --- |
|  |  | LL | UL |  |
| Dutch |  |  |  |  |
| Emotional neglect | 1.31 | 1.12 | 1.55 | .001 |
| Age | 0.97 | 0.97 | 0.98 | < .001 |
| Sex: Female | 0.34 | 0.30 | 0.40 | < .001 |
| Education level: low-medium | 0.84 | 0.53 | 1.33 | .457 |
| Education level: medium-high | 1.09 | 0.70 | 1.70 | .696 |
| Education level: high | 1.24 | 0.81 | 1.89 | .326 |
| Parental alcohol misuse | 1.18 | 0.94 | 1.49 | .156 |
| South-Asian Surinamese |  |  |  |  |
| Emotional neglect | 1.78 | 1.33 | 2.39 | < .001 |
| Age | 0.98 | 0.97 | 0.99 | < .001 |
| Sex: Female | 0.13 | 0.09 | 0.18 | < .001 |
| Education level: low-medium | 0.81 | 0.54 | 1.22 | .318 |
| Education level: medium-high | 0.57 | 0.37 | 0.88 | .012 |
| Education level: high | 0.69 | 0.44 | 1.07 | .099 |
| Parental alcohol misuse | 1.95 | 1.45 | 2.64 | < .001 |
| African Surinamese |  |  |  |  |
| Emotional neglect | 1.75 | 1.34 | 2.29 | < .001 |
| Age | 0.98 | 0.97 | 0.99 | < .001 |
| Sex: Female | 0.21 | 0.16 | 0.27 | < .001 |
| Education level: low-medium | 0.63 | 0.40 | 0.97 | .038 |
| Education level: medium-high | 0.47 | 0.29 | 0.74 | .001 |
| Education level: high | 0.42 | 0.26 | 0.70 | .001 |
| Parental alcohol misuse | 1.96 | 1.39 | 2.76 | < .001 |
| Ghanaian |  |  |  |  |
| Emotional neglect | 1.77 | 1.15 | 2.70 | .009 |
| Age | 1.01 | 0.99 | 1.02 | .317 |
| Sex: Female | 0.31 | 0.21 | 0.44 | < .001 |
| Education level: low-medium | 1.09 | 0.70 | 1.69 | .714 |
| Education level: medium-high | 0.95 | 0.58 | 1.57 | .846 |
| Education level: high | 0.53 | 0.21 | 1.31 | .168 |
| Parental alcohol misuse | 3.00 | 1.71 | 5.24 | < .001 |
| Turkish |  |  |  |  |
| Emotional neglect | 1.77 | 1.23 | 2.54 | .002 |
| Age | 0.99 | 0.98 | 1.01 | .428 |
| Sex: Female | 0.07 | 0.04 | 0.12 | < .001 |
| Education level: low-medium | 0.80 | 0.50 | 1.28 | .362 |
| Education level: medium-high | 0.93 | 0.58 | 1.51 | .777 |
| Education level: high | 0.86 | 0.50 | 1.50 | .603 |
| Parental alcohol misuse | 3.47 | 2.02 | 5.96 | < .001 |
| Moroccan |  |  |  |  |
| Emotional neglect | 3.05 | 1.97 | 4.73 | < .001 |
| Age | 0.97 | 0.95 | 0.99 | .004 |
| Sex: Female | 0.10 | 0.06 | 0.18 | < .001 |
| Education level: low-medium | 1.53 | 0.78 | 3.01 | .219 |
| Education level: medium-high | 1.04 | 0.53 | 2.04 | .908 |
| Education level: high | 1.73 | 0.87 | 3.44 | .117 |
| Parental alcohol misuse | 3.67 | 1.61 | 8.36 | .002 |

*Note.* AUDIT = alcohol use disorder identification test; CI = confidence interval; LL = lower level; OR = odds ratio; UL = upper level.

Reference group for education level = low.
Pooled results based on multiple imputed datasets.

**Supplementary Table S5b**

Association between psychological abuse and problematic alcohol use (AUDIT ≥ 8) per ethnicity, adjusted for confounding by age, sex, education level, and parental alcohol misuse.

|  | OR | 95% CI for OR | | *P* |
| --- | --- | --- | --- | --- |
|  |  | LL | UL |  |
| Dutch |  |  |  |  |
| Psychological abuse | 1.05 | 0.86 | 1.28 | .654 |
| Age | 0.97 | 0.97 | 0.98 | < .001 |
| Sex: Female | 0.35 | 0.30 | 0.40 | < .001 |
| Education level: low-medium | 0.83 | 0.53 | 1.32 | .434 |
| Education level: medium-high | 1.11 | 0.72 | 1.72 | .644 |
| Education level: high | 1.26 | 0.82 | 1.91 | .292 |
| Parental alcohol misuse | 1.28 | 1.02 | 1.60 | .035 |
| South-Asian Surinamese |  |  |  |  |
| Psychological abuse | 1.67 | 1.22 | 2.29 | .002 |
| Age | 0.98 | 0.97 | 0.99 | < .001 |
| Sex: Female | 0.13 | 0.09 | 0.18 | < .001 |
| Education level: low-medium | 0.80 | 0.54 | 1.20 | .287 |
| Education level: medium-high | 0.56 | 0.36 | 0.87 | .011 |
| Education level: high | 0.70 | 0.45 | 1.08 | .108 |
| Parental alcohol misuse | 2.00 | 1.48 | 2.70 | < .001 |
| African Surinamese |  |  |  |  |
| Psychological abuse | 1.71 | 1.28 | 2.29 | < .001 |
| Age | 0.98 | 0.97 | 0.99 | < .001 |
| Sex: Female | 0.21 | 0.16 | 0.28 | < .001 |
| Education level: low-medium | 0.64 | 0.41 | 1.00 | .048 |
| Education level: medium-high | 0.48 | 0.30 | 0.76 | .002 |
| Education level: high | 0.44 | 0.27 | 0.73 | .001 |
| Parental alcohol misuse | 2.01 | 1.43 | 2.82 | < .001 |
| Ghanaian |  |  |  |  |
| Psychological abuse | 1.52 | 0.93 | 2.50 | .099 |
| Age | 1.01 | 0.99 | 1.02 | .338 |
| Sex: Female | 0.31 | 0.22 | 0.45 | < .001 |
| Education level: low-medium | 1.08 | 0.70 | 1.69 | .726 |
| Education level: medium-high | 0.95 | 0.58 | 1.57 | .851 |
| Education level: high | 0.53 | 0.21 | 1.30 | .166 |
| Parental alcohol misuse | 3.07 | 1.73 | 5.44 | < .001 |
| Turkish |  |  |  |  |
| Psychological abuse | 1.97 | 1.28 | 3.01 | .002 |
| Age | 0.99 | 0.98 | 1.01 | .487 |
| Sex: Female | 0.07 | 0.04 | 0.12 | < .001 |
| Education level: low-medium | 0.79 | 0.50 | 1.26 | .325 |
| Education level: medium-high | 0.91 | 0.56 | 1.47 | .695 |
| Education level: high | 0.83 | 0.48 | 1.45 | .523 |
| Parental alcohol misuse | 3.41 | 1.98 | 5.87 | < .001 |
| Moroccan |  |  |  |  |
| Psychological abuse | 3.22 | 2.03 | 5.11 | < .001 |
| Age | 0.98 | 0.96 | 0.99 | .010 |
| Sex: Female | 0.10 | 0.06 | 0.18 | < .001 |
| Education level: low-medium | 1.63 | 0.83 | 3.19 | .159 |
| Education level: medium-high | 1.12 | 0.57 | 2.20 | .743 |
| Education level: high | 1.78 | 0.90 | 3.53 | .099 |
| Parental alcohol misuse | 4.09 | 1.83 | 9.16 | .001 |

*Note.* AUDIT = alcohol use disorder identification test; CI = confidence interval; LL = lower level; OR = odds ratio; UL = upper level.

Reference group for education level = low.
Pooled results based on multiple imputed datasets.

**Supplementary Table S5c**

Association between physical abuse and problematic alcohol use (AUDIT ≥ 8) per ethnicity, adjusted for confounding by age, sex, education level, and parental alcohol misuse.

|  | OR | 95% CI for OR | | *P* |
| --- | --- | --- | --- | --- |
|  |  | LL | UL |  |
| Dutch |  |  |  |  |
| Physical abuse | 1.16 | 0.91 | 1.47 | .228 |
| Age | 0.97 | 0.97 | 0.98 | < .001 |
| Sex: Female | 0.35 | 0.30 | 0.40 | < .001 |
| Education level: low-medium | 0.84 | 0.53 | 1.32 | .447 |
| Education level: medium-high | 1.11 | 0.72 | 1.73 | .632 |
| Education level: high | 1.26 | 0.83 | 1.93 | .279 |
| Parental alcohol misuse | 1.27 | 1.02 | 1.59 | .036 |
| South-Asian Surinamese |  |  |  |  |
| Physical abuse | 1.92 | 1.43 | 2.59 | < .001 |
| Age | 0.98 | 0.97 | 0.99 | < .001 |
| Sex: Female | 0.13 | 0.09 | 0.18 | < .001 |
| Education level: low-medium | 0.81 | 0.54 | 1.21 | .294 |
| Education level: medium-high | 0.55 | 0.36 | 0.86 | .009 |
| Education level: high | 0.69 | 0.44 | 1.06 | .092 |
| Parental alcohol misuse | 1.99 | 1.48 | 2.66 | < .001 |
| African Surinamese |  |  |  |  |
| Physical abuse | 1.53 | 1.17 | 2.01 | .002 |
| Age | 0.98 | 0.97 | 0.99 | < .001 |
| Sex: Female | 0.22 | 0.17 | 0.28 | < .001 |
| Education level: low-medium | 0.63 | 0.41 | 0.98 | .038 |
| Education level: medium-high | 0.47 | 0.30 | 0.74 | .001 |
| Education level: high | 0.43 | 0.26 | 0.71 | .001 |
| Parental alcohol misuse | 2.08 | 1.48 | 2.91 | < .001 |
| Ghanaian |  |  |  |  |
| Physical abuse | 2.24 | 1.52 | 3.31 | < .001 |
| Age | 1.01 | 0.99 | 1.02 | .341 |
| Sex: Female | 0.31 | 0.22 | 0.45 | < .001 |
| Education level: low-medium | 1.07 | 0.69 | 1.67 | .756 |
| Education level: medium-high | 0.92 | 0.56 | 1.52 | .746 |
| Education level: high | 0.49 | 0.20 | 1.22 | .125 |
| Parental alcohol misuse | 2.90 | 1.65 | 5.11 | < .001 |
| Turkish |  |  |  |  |
| Physical abuse | 2.41 | 1.62 | 3.58 | < .001 |
| Age | 0.99 | 0.98 | 1.01 | .333 |
| Sex: Female | 0.07 | 0.04 | 0.13 | < .001 |
| Education level: low-medium | 0.76 | 0.48 | 1.22 | .259 |
| Education level: medium-high | 0.88 | 0.54 | 1.43 | .614 |
| Education level: high | 0.77 | 0.44 | 1.35 | .364 |
| Parental alcohol misuse | 3.39 | 1.97 | 5.84 | < .001 |
| Moroccan |  |  |  |  |
| Physical abuse | 4.64 | 3.03 | 7.12 | < .001 |
| Age | 0.97 | 0.95 | 0.99 | .006 |
| Sex: Female | 0.11 | 0.07 | 0.19 | < .001 |
| Education level: low-medium | 1.65 | 0.84 | 3.25 | .146 |
| Education level: medium-high | 1.13 | 0.57 | 2.23 | .728 |
| Education level: high | 1.69 | 0.85 | 3.36 | .136 |
| Parental alcohol misuse | 3.46 | 1.50 | 7.94 | .004 |

*Note.* AUDIT = alcohol use disorder identification test; CI = confidence interval; LL = lower level; OR = odds ratio; UL = upper level.

Reference group for education level = low.
Pooled results based on multiple imputed datasets.

**Supplementary Table S5d**

Association between sexual abuse and problematic alcohol use (AUDIT ≥ 8) per ethnicity, adjusted for confounding by age, sex, education level, and parental alcohol misuse.

|  | OR | 95% CI for OR | | *P* |
| --- | --- | --- | --- | --- |
|  |  | LL | UL |  |
| Dutch |  |  |  |  |
| Sexual abuse | 1.39 | 1.11 | 1.75 | .004 |
| Age | 0.97 | 0.97 | 0.98 | < .001 |
| Sex: Female | 0.34 | 0.29 | 0.39 | < .001 |
| Education level: low-medium | 0.83 | 0.53 | 1.32 | .437 |
| Education level: medium-high | 1.11 | 0.72 | 1.72 | .638 |
| Education level: high | 1.26 | 0.82 | 1.92 | .286 |
| Parental alcohol misuse | 1.26 | 1.01 | 1.57 | .042 |
| South-Asian Surinamese |  |  |  |  |
| Sexual abuse | 2.75 | 1.65 | 4.56 | < .001 |
| Age | 0.98 | 0.97 | 0.99 | < .001 |
| Sex: Female | 0.11 | 0.08 | 0.16 | < .001 |
| Education level: low-medium | 0.81 | 0.54 | 1.21 | .294 |
| Education level: medium-high | 0.56 | 0.36 | 0.87 | .009 |
| Education level: high | 0.69 | 0.44 | 1.06 | .093 |
| Parental alcohol misuse | 2.13 | 1.59 | 2.85 | < .001 |
| African Surinamese |  |  |  |  |
| Sexual abuse | 1.44 | 0.96 | 2.16 | .082 |
| Age | 0.98 | 0.97 | 0.99 | < .001 |
| Sex: Female | 0.21 | 0.16 | 0.27 | < .001 |
| Education level: low-medium | 0.63 | 0.41 | 0.98 | .043 |
| Education level: medium-high | 0.47 | 0.29 | 0.74 | .001 |
| Education level: high | 0.43 | 0.26 | 0.71 | .001 |
| Parental alcohol misuse | 2.14 | 1.54 | 2.99 | < .001 |
| Ghanaian |  |  |  |  |
| Sexual abuse | 1.71 | 0.92 | 3.19 | .092 |
| Age | 1.01 | 0.99 | 1.02 | .332 |
| Sex: Female | 0.31 | 0.21 | 0.44 | < .001 |
| Education level: low-medium | 1.06 | 0.68 | 1.66 | .783 |
| Education level: medium-high | 0.93 | 0.57 | 1.54 | .790 |
| Education level: high | 0.50 | 0.20 | 1.26 | .143 |
| Parental alcohol misuse | 3.22 | 1.84 | 5.62 | < .001 |
| Turkish |  |  |  |  |
| Sexual abuse | 3.16 | 1.46 | 6.84 | .004 |
| Age | 1.00 | 0.98 | 1.01 | .541 |
| Sex: Female | 0.07 | 0.04 | 0.12 | < .001 |
| Education level: low-medium | 0.78 | 0.49 | 1.25 | .308 |
| Education level: medium-high | 0.90 | 0.56 | 1.47 | .685 |
| Education level: high | 0.84 | 0.48 | 1.46 | .537 |
| Parental alcohol misuse | 3.92 | 2.29 | 6.70 | < .001 |
| Moroccan |  |  |  |  |
| Sexual abuse | 5.02 | 2.78 | 9.07 | < .001 |
| Age | 0.97 | 0.95 | 0.99 | .004 |
| Sex: Female | 0.10 | 0.06 | 0.17 | < .001 |
| Education level: low-medium | 1.46 | 0.74 | 2.88 | .270 |
| Education level: medium-high | 1.02 | 0.52 | 2.00 | .961 |
| Education level: high | 1.55 | 0.78 | 3.09 | .210 |
| Parental alcohol misuse | 3.74 | 1.62 | 8.61 | .002 |

*Note.* AUDIT = alcohol use disorder identification test; CI = confidence interval; LL = lower level; OR = odds ratio; UL = upper level.

Reference group for education level = low.
Pooled results based on multiple imputed datasets.

**Supplementary Table S6**

Associations between child maltreatment types and problematic alcohol use (AUDIT ≥ 8) per ethnicity, adjusted for confounding by age, sex, education level, and parental alcohol misuse.

|  | OR | 95% CI for OR | | *P* |
| --- | --- | --- | --- | --- |
|  |  | LL | UL |  |
| Dutch |  |  |  |  |
| Emotional neglect | 1.37 | 1.13 | 1.65 | .001 |
| Psychological abuse | 0.80 | 0.62 | 1.02 | .075 |
| Physical abuse | 1.09 | 0.83 | 1.44 | .518 |
| Sexual abuse | 1.33 | 1.05 | 1.68 | .017 |
| Age | 0.97 | 0.97 | 0.98 | < .001 |
| Sex: Female | 0.33 | 0.29 | 0.38 | < .001 |
| Education level: low-medium | 0.85 | 0.54 | 1.35 | .488 |
| Education level: medium-high | 1.10 | 0.71 | 1.71 | .669 |
| Education level: high | 1.25 | 0.81 | 1.90 | .312 |
| Parental alcohol misuse | 1.19 | 0.94 | 1.50 | .144 |
| South-Asian Surinamese |  |  |  |  |
| Emotional neglect | 1.44 | 0.97 | 2.14 | .069 |
| Psychological abuse | 0.88 | 0.55 | 1.41 | .591 |
| Physical abuse | 1.56 | 1.05 | 2.31 | .027 |
| Sexual abuse | 2.12 | 1.25 | 3.59 | .005 |
| Age | 0.98 | 0.97 | 0.99 | < .001 |
| Sex: Female | 0.11 | 0.08 | 0.16 | < .001 |
| Education level: low-medium | 0.80 | 0.53 | 1.21 | .291 |
| Education level: medium-high | 0.55 | 0.35 | 0.86 | .008 |
| Education level: high | 0.67 | 0.43 | 1.04 | .074 |
| Parental alcohol misuse | 1.85 | 1.37 | 2.52 | < .001 |
| African Surinamese |  |  |  |  |
| Emotional neglect | 1.46 | 1.00 | 2.14 | .049 |
| Psychological abuse | 1.20 | 0.76 | 1.89 | .436 |
| Physical abuse | 1.10 | 0.77 | 1.59 | .598 |
| Sexual abuse | 1.11 | 0.72 | 1.71 | .638 |
| Age | 0.98 | 0.97 | 0.99 | < .001 |
| Sex: Female | 0.20 | 0.15 | 0.27 | < .001 |
| Education level: low-medium | 0.63 | 0.41 | 0.98 | .041 |
| Education level: medium-high | 0.47 | 0.30 | 0.74 | .001 |
| Education level: high | 0.43 | 0.26 | 0.70 | .001 |
| Parental alcohol misuse | 1.92 | 1.36 | 2.70 | < .001 |
| Ghanaian |  |  |  |  |
| Emotional neglect | 1.41 | 0.79 | 2.49 | .246 |
| Psychological abuse | 0.65 | 0.32 | 1.31 | .228 |
| Physical abuse | 2.24 | 1.37 | 3.65 | .001 |
| Sexual abuse | 1.40 | 0.72 | 2.70 | .318 |
| Age | 1.01 | 0.99 | 1.02 | .324 |
| Sex: Female | 0.31 | 0.21 | 0.44 | < .001 |
| Education level: low-medium | 1.06 | 0.68 | 1.66 | .782 |
| Education level: medium-high | 0.91 | 0.55 | 1.50 | .708 |
| Education level: high | 0.47 | 0.19 | 1.19 | .112 |
| Parental alcohol misuse | 2.88 | 1.62 | 5.11 | < .001 |
| Turkish |  |  |  |  |
| Emotional neglect | 1.25 | 0.79 | 1.99 | .345 |
| Psychological abuse | 1.08 | 0.61 | 1.92 | .781 |
| Physical abuse | 1.92 | 1.17 | 3.14 | .009 |
| Sexual abuse | 2.12 | 0.94 | 4.78 | .070 |
| Age | 0.99 | 0.98 | 1.01 | .302 |
| Sex: Female | 0.07 | 0.04 | 0.12 | < .001 |
| Education level: low-medium | 0.77 | 0.48 | 1.23 | .267 |
| Education level: medium-high | 0.89 | 0.54 | 1.45 | .633 |
| Education level: high | 0.76 | 0.43 | 1.34 | .345 |
| Parental alcohol misuse | 3.17 | 1.81 | 5.53 | < .001 |
| Moroccan |  |  |  |  |
| Emotional neglect | 1.29 | 0.69 | 2.40 | .420 |
| Psychological abuse | 1.06 | 0.54 | 2.05 | .873 |
| Physical abuse | 3.30 | 1.86 | 5.85 | < .001 |
| Sexual abuse | 2.54 | 1.32 | 4.90 | .005 |
| Age | 0.97 | 0.95 | 0.99 | .003 |
| Sex: Female | 0.10 | 0.06 | 0.18 | < .001 |
| Education level: low-medium | 1.56 | 0.79 | 3.09 | .202 |
| Education level: medium-high | 1.10 | 0.56 | 2.17 | .783 |
| Education level: high | 1.58 | 0.79 | 3.14 | .196 |
| Parental alcohol misuse | 2.89 | 1.23 | 6.81 | .015 |

*Note.* AUDIT = alcohol use disorder identification test; CI = confidence interval; LL = lower level; OR = odds ratio; UL = upper level.

For each ethnicity, all types of child maltreatment were included in the same model.

Reference group for education level = low.
Pooled results based on multiple imputed datasets.

**Supplementary Table S7**

Correlation matrix showing correlations (phi-coefficients) between types of child maltreatment in the total sample and per ethnicity.

|  | 1 | 2 | 3 | 4 |
| --- | --- | --- | --- | --- |
| Total sample |  |  |  |  |
| 1. Emotional neglect | - |  |  |  |
| 2. Psychological abuse | .61 | - |  |  |
| 3. Physical abuse | .44 | .57 | - |  |
| 4. Sexual abuse | .26 | .27 | .24 | - |
| Dutch |  |  |  |  |
| 1. Emotional neglect | - |  |  |  |
| 2. Psychological abuse | .56 | - |  |  |
| 3. Physical abuse | .33 | .49 | - |  |
| 4. Sexual abuse | .24 | .23 | .20 | - |
| South-Asian Surinamese |  |  |  |  |
| 1. Emotional neglect | - |  |  |  |
| 2. Psychological abuse | .66 | - |  |  |
| 3. Physical abuse | .50 | .61 | - |  |
| 4. Sexual abuse | .26 | .26 | .24 | - |
| African Surinamese |  |  |  |  |
| 1. Emotional neglect | - |  |  |  |
| 2. Psychological abuse | .66 | - |  |  |
| 3. Physical abuse | .51 | .62 | - |  |
| 4. Sexual abuse | .33 | .31 | .29 | - |
| Ghanaian |  |  |  |  |
| 1. Emotional neglect | - |  |  |  |
| 2. Psychological abuse | .62 | - |  |  |
| 3. Physical abuse | .47 | .56 | - |  |
| 4. Sexual abuse | .23 | .30 | .23 | - |
| Turkish |  |  |  |  |
| 1. Emotional neglect | - |  |  |  |
| 2. Psychological abuse | .53 | - |  |  |
| 3. Physical abuse | .42 | .56 | - |  |
| 4. Sexual abuse | .19 | .22 | .21 | - |
| Moroccan |  |  |  |  |
| 1. Emotional neglect | - |  |  |  |
| 2. Psychological abuse | .63 | - |  |  |
| 3. Physical abuse | .49 | .59 | - |  |
| 4. Sexual abuse | .27 | .26 | .28 | - |

*Note.* Chi-square tests indicated that all correlations are statistically significant at the level of *p* < .001.

**Supplementary Table S8**

The association between (any) child maltreatment and problematic alcohol use (AUDIT ≥ 8 for males, AUDIT ≥ 6 for females), adjusted for confounding by age, sex, education level, parental alcohol misuse, and ethnicity.

|  | OR | 95% CI for OR | | *P* |
| --- | --- | --- | --- | --- |
|  |  | LL | UL |  |
| Any child maltreatment | 1.56 | 1.42 | 1.71 | < .001 |
| Age | 0.98 | 0.97 | 0.98 | < .001 |
| Sex: Female | 0.47 | 0.43 | 0.51 | < .001 |
| Education level: low-medium | 0.90 | 0.76 | 1.07 | .252 |
| Education level: medium-high | 0.82 | 0.69 | 0.97 | .024 |
| Education level: high | 1.02 | 0.85 | 1.21 | .857 |
| Parental alcohol misuse | 1.61 | 1.40 | 1.84 | < .001 |
| Ethnicity: South-Asian Surinamese | 0.19 | 0.17 | 0.22 | < .001 |
| Ethnicity: African Surinamese | 0.20 | 0.18 | 0.23 | < .001 |
| Ethnicity: Ghanaian | 0.18 | 0.15 | 0.21 | < .001 |
| Ethnicity: Turkish | 0.09 | 0.07 | 0.10 | < .001 |
| Ethnicity: Moroccan | 0.05 | 0.04 | 0.07 | < .001 |

*Note.* AUDIT = alcohol use disorder identification test; CI = confidence interval; LL = lower level; OR = odds ratio; UL = upper level.

Reference group for education level = low; reference group for ethnicity = Dutch.

Pooled results based on multiple imputed datasets.

**Supplementary Table S9**

Effect modification of ethnicity on the association between (any) child maltreatment and problematic alcohol use (AUDIT ≥ 8 for males, AUDIT ≥ 6 for females), adjusted for confounding by age, sex, education level, parental alcohol misuse, and ethnicity.

|  | OR | 95% CI for OR | | *P* |
| --- | --- | --- | --- | --- |
|  |  | LL | UL |  |
| Any child maltreatment | 1.17 | 1.02 | 1.34 | .021 |
| Age | 0.98 | 0.97 | 0.98 | < .001 |
| Sex: Female | 0.47 | 0.43 | 0.52 | < .001 |
| Education level: low-medium | 0.90 | 0.76 | 1.07 | .232 |
| Education level: medium-high | 0.82 | 0.69 | 0.97 | .024 |
| Education level: high | 1.01 | 0.84 | 1.20 | .948 |
| Parental alcohol misuse | 1.63 | 1.42 | 1.86 | < .001 |
| Ethnicity: South-Asian Surinamese | 0.17 | 0.14 | 0.20 | < .001 |
| Ethnicity: African Surinamese | 0.18 | 0.15 | 0.21 | < .001 |
| Ethnicity: Ghanaian | 0.14 | 0.11 | 0.18 | < .001 |
| Ethnicity: Turkish | 0.07 | 0.05 | 0.08 | < .001 |
| Ethnicity: Moroccan | 0.03 | 0.03 | 0.04 | < .001 |
| Any child maltreatment * South-Asian Surinamese | 1.49 | 1.13 | 1.96 | .004 |
| Any child maltreatment * African Surinamese | 1.38 | 1.07 | 1.77 | .013 |
| Any child maltreatment * Ghanaian | 1.87 | 1.33 | 2.63 | < .001 |
| Any child maltreatment * Turkish | 1.99 | 1.43 | 2.76 | < .001 |
| Any child maltreatment * Moroccan | 3.26 | 2.18 | 4.88 | < .001 |

*Note.* AUDIT = alcohol use disorder identification test; CI = confidence interval; LL = lower level; OR = odds ratio; UL = upper level.

Reference group for education level = low; reference group for ethnicity = Dutch.
Pooled results based on multiple imputed datasets.

**Supplementary Table S10**

Association between (any) child maltreatment and problematic alcohol use (AUDIT ≥ 8 for males, AUDIT ≥ 6 for females) per ethnicity, adjusted for confounding by age, sex, education level, and parental alcohol misuse.

|  | OR | 95% CI for OR | | *P* |
| --- | --- | --- | --- | --- |
|  |  | LL | UL |  |
| Dutch |  |  |  |  |
| Any child maltreatment | 1.19 | 1.04 | 1.37 | .011 |
| Age | 0.97 | 0.97 | 0.98 | < .001 |
| Sex: Female | 0.77 | 0.68 | 0.87 | < .001 |
| Education level: low-medium | 0.80 | 0.53 | 1.21 | .288 |
| Education level: medium-high | 1.08 | 0.73 | 1.61 | .697 |
| Education level: high | 1.32 | 0.90 | 1.94 | .154 |
| Parental alcohol misuse | 1.17 | 0.96 | 1.43 | .120 |
| South-Asian Surinamese |  |  |  |  |
| Any child maltreatment | 1.82 | 1.41 | 2.34 | < .001 |
| Age | 0.97 | 0.96 | 0.98 | < .001 |
| Sex: Female | 0.23 | 0.18 | 0.31 | < .001 |
| Education level: low-medium | 0.76 | 0.52 | 1.11 | .156 |
| Education level: medium-high | 0.53 | 0.35 | 0.80 | .003 |
| Education level: high | 0.66 | 0.44 | 0.99 | .045 |
| Parental alcohol misuse | 2.01 | 1.53 | 2.65 | < .001 |
| African Surinamese |  |  |  |  |
| Any child maltreatment | 1.69 | 1.35 | 2.11 | < .001 |
| Age | 0.98 | 0.97 | 0.99 | < .001 |
| Sex: Female | 0.41 | 0.32 | 0.51 | < .001 |
| Education level: low-medium | 0.65 | 0.43 | 0.98 | .038 |
| Education level: medium-high | 0.47 | 0.31 | 0.72 | < .001 |
| Education level: high | 0.41 | 0.26 | 0.65 | < .001 |
| Parental alcohol misuse | 1.75 | 1.28 | 2.39 | < .001 |
| Ghanaian |  |  |  |  |
| Any child maltreatment | 2.24 | 1.63 | 3.08 | < .001 |
| Age | 1.01 | 1.00 | 1.02 | .189 |
| Sex: Female | 0.55 | 0.40 | 0.76 | < .001 |
| Education level: low-medium | 1.27 | 0.85 | 1.91 | .250 |
| Education level: medium-high | 1.19 | 0.76 | 1.86 | .454 |
| Education level: high | 0.50 | 0.20 | 1.21 | .124 |
| Parental alcohol misuse | 2.39 | 1.41 | 4.08 | .001 |
| Turkish |  |  |  |  |
| Any child maltreatment | 2.19 | 1.59 | 3.01 | < .001 |
| Age | 0.99 | 0.98 | 1.00 | .170 |
| Sex: Female | 0.16 | 0.11 | 0.24 | < .001 |
| Education level: low-medium | 0.84 | 0.53 | 1.34 | .467 |
| Education level: medium-high | 1.04 | 0.66 | 1.65 | .869 |
| Education level: high | 1.13 | 0.68 | 1.88 | .628 |
| Parental alcohol misuse | 3.22 | 1.93 | 5.38 | < .001 |
| Moroccan |  |  |  |  |
| Any child maltreatment | 3.53 | 2.39 | 5.23 | < .001 |
| Age | 0.97 | 0.96 | 0.99 | .006 |
| Sex: Female | 0.17 | 0.11 | 0.26 | < .001 |
| Education level: low-medium | 1.62 | 0.83 | 3.16 | .157 |
| Education level: medium-high | 1.25 | 0.65 | 2.40 | .512 |
| Education level: high | 1.78 | 0.90 | 3.50 | .097 |
| Parental alcohol misuse | 4.01 | 1.81 | 8.90 | .001 |

*Note.* AUDIT = alcohol use disorder identification test; CI = confidence interval; LL = lower level; OR = odds ratio; UL = upper level.

Reference group for education level = low.
Pooled results based on multiple imputed datasets.

**Supplementary Table S11**

Effect modification of ethnicity on the associations between specific types of child maltreatment and problematic alcohol use (AUDIT ≥ 8 for males, AUDIT ≥ 6 for females), adjusted for confounding by age, sex, education level, parental alcohol misuse, and ethnicity.

|  | OR | 95% CI for OR | | *P* |
| --- | --- | --- | --- | --- |
|  |  | LL | UL |  |
| Emotional neglect | 1.19 | 1.03 | 1.38 | .017 |
| Age | 0.98 | 0.97 | 0.98 | < .001 |
| Sex: Female | 0.47 | 0.43 | 0.52 | < .001 |
| Education level: low-medium | 0.91 | 0.77 | 1.08 | .294 |
| Education level: medium-high | 0.83 | 0.70 | 0.99 | .043 |
| Education level: high | 1.03 | 0.87 | 1.23 | .724 |
| Parental alcohol misuse | 1.63 | 1.42 | 1.87 | < .001 |
| Ethnicity: South-Asian Surinamese | 0.18 | 0.15 | 0.21 | < .001 |
| Ethnicity: African Surinamese | 0.19 | 0.16 | 0.22 | < .001 |
| Ethnicity: Ghanaian | 0.16 | 0.13 | 0.19 | < .001 |
| Ethnicity: Turkish | 0.07 | 0.06 | 0.09 | < .001 |
| Ethnicity: Moroccan | 0.04 | 0.03 | 0.05 | < .001 |
| Emotional neglect * South-Asian Surinamese | 1.41 | 1.05 | 1.88 | .020 |
| Emotional neglect * African Surinamese | 1.44 | 1.10 | 1.90 | .008 |
| Emotional neglect * Ghanaian | 1.65 | 1.11 | 2.45 | .013 |
| Emotional neglect * Turkish | 1.62 | 1.15 | 2.29 | .006 |
| Emotional neglect * Moroccan | 2.93 | 1.93 | 4.46 | < .001 |
| Psychological abuse | 0.99 | 0.83 | 1.18 | .916 |
| Age | 0.98 | 0.97 | 0.98 | < .001 |
| Sex: Female | 0.48 | 0.44 | 0.52 | < .001 |
| Education level: low-medium | 0.91 | 0.76 | 1.08 | .283 |
| Education level: medium-high | 0.84 | 0.70 | 1.00 | .049 |
| Education level: high | 1.04 | 0.87 | 1.24 | .664 |
| Parental alcohol misuse | 1.70 | 1.48 | 1.94 | < .001 |
| Ethnicity: South-Asian Surinamese | 0.18 | 0.15 | 0.21 | < .001 |
| Ethnicity: African Surinamese | 0.18 | 0.16 | 0.21 | < .001 |
| Ethnicity: Ghanaian | 0.17 | 0.14 | 0.20 | < .001 |
| Ethnicity: Turkish | 0.08 | 0.06 | 0.09 | < .001 |
| Ethnicity: Moroccan | 0.04 | 0.03 | 0.05 | < .001 |
| Psychological abuse * South-Asian Surinamese | 1.56 | 1.13 | 2.15 | .007 |
| Psychological abuse * African Surinamese | 1.71 | 1.26 | 2.33 | .001 |
| Psychological abuse * Ghanaian | 1.51 | 0.94 | 2.42 | .086 |
| Psychological abuse * Turkish | 2.16 | 1.43 | 3.26 | < .001 |
| Psychological abuse * Moroccan | 3.80 | 2.43 | 5.95 | < .001 |
| Physical abuse | 1.02 | 0.82 | 1.27 | .840 |
| Age | 0.98 | 0.97 | 0.98 | < .001 |
| Sex: Female | 0.49 | 0.45 | 0.53 | < .001 |
| Education level: low-medium | 0.90 | 0.76 | 1.07 | .246 |
| Education level: medium-high | 0.83 | 0.70 | 0.99 | .036 |
| Education level: high | 1.02 | 0.85 | 1.21 | .840 |
| Parental alcohol misuse | 1.68 | 1.47 | 1.92 | < .001 |
| Ethnicity: South-Asian Surinamese | 0.17 | 0.15 | 0.20 | < .001 |
| Ethnicity: African Surinamese | 0.18 | 0.16 | 0.21 | < .001 |
| Ethnicity: Ghanaian | 0.15 | 0.12 | 0.18 | < .001 |
| Ethnicity: Turkish | 0.07 | 0.06 | 0.09 | < .001 |
| Ethnicity: Moroccan | 0.03 | 0.02 | 0.04 | < .001 |
| Physical abuse * South-Asian Surinamese | 1.69 | 1.19 | 2.39 | .003 |
| Physical abuse * African Surinamese | 1.55 | 1.12 | 2.14 | .008 |
| Physical abuse * Ghanaian | 2.14 | 1.42 | 3.24 | < .001 |
| Physical abuse * Turkish | 2.70 | 1.79 | 4.09 | < .001 |
| Physical abuse * Moroccan | 5.58 | 3.55 | 8.77 | < .001 |
| Sexual abuse | 1.49 | 1.22 | 1.81 | < .001 |
| Age | 0.98 | 0.97 | 0.98 | < .001 |
| Sex: Female | 0.46 | 0.42 | 0.50 | < .001 |
| Education level: low-medium | 0.89 | 0.75 | 1.06 | .195 |
| Education level: medium-high | 0.82 | 0.69 | 0.97 | .024 |
| Education level: high | 1.02 | 0.85 | 1.21 | .863 |
| Parental alcohol misuse | 1.73 | 1.52 | 1.98 | < .001 |
| Ethnicity: South-Asian Surinamese | 0.19 | 0.17 | 0.23 | < .001 |
| Ethnicity: African Surinamese | 0.21 | 0.18 | 0.24 | < .001 |
| Ethnicity: Ghanaian | 0.17 | 0.14 | 0.21 | < .001 |
| Ethnicity: Turkish | 0.08 | 0.07 | 0.10 | < .001 |
| Ethnicity: Moroccan | 0.05 | 0.04 | 0.06 | < .001 |
| Sexual abuse * South-Asian Surinamese | 1.14 | 0.72 | 1.80 | .574 |
| Sexual abuse * African Surinamese | 0.93 | 0.64 | 1.35 | .690 |
| Sexual abuse * Ghanaian | 1.24 | 0.71 | 2.15 | .446 |
| Sexual abuse * Turkish | 2.37 | 1.27 | 4.43 | .007 |
| Sexual abuse * Moroccan | 3.63 | 2.11 | 6.27 | < .001 |

*Note.* AUDIT = alcohol use disorder identification test; CI = confidence interval; LL = lower level; OR = odds ratio; UL = upper level.

Reference group for education level = low; reference group for ethnicity = Dutch.
Pooled results based on multiple imputed datasets.

**Supplementary Table S12a**

Association between emotional neglect and problematic alcohol use (AUDIT ≥ 8 for males, AUDIT ≥ 6 for females) per ethnicity, adjusted for confounding by age, sex, education level, and parental alcohol misuse.

|  | OR | 95% CI for OR | | *P* |
| --- | --- | --- | --- | --- |
|  |  | LL | UL |  |
| Dutch |  |  |  |  |
| Emotional neglect | 1.24 | 1.07 | 1.44 | .004 |
| Age | 0.97 | 0.97 | 0.98 | < .001 |
| Sex: Female | 0.77 | 0.68 | 0.87 | < .001 |
| Education level: low-medium | 0.80 | 0.53 | 1.21 | .286 |
| Education level: medium-high | 1.08 | 0.72 | 1.60 | .714 |
| Education level: high | 1.31 | 0.90 | 1.93 | .163 |
| Parental alcohol misuse | 1.16 | 0.94 | 1.42 | .163 |
| South-Asian Surinamese |  |  |  |  |
| Emotional neglect | 1.70 | 1.30 | 2.23 | < .001 |
| Age | 0.97 | 0.96 | 0.98 | < .001 |
| Sex: Female | 0.24 | 0.18 | 0.31 | < .001 |
| Education level: low-medium | 0.77 | 0.53 | 1.13 | .178 |
| Education level: medium-high | 0.54 | 0.35 | 0.81 | .003 |
| Education level: high | 0.67 | 0.45 | 1.01 | .059 |
| Parental alcohol misuse | 2.05 | 1.56 | 2.71 | < .001 |
| African Surinamese |  |  |  |  |
| Emotional neglect | 1.78 | 1.40 | 2.27 | < .001 |
| Age | 0.98 | 0.97 | 0.99 | < .001 |
| Sex: Female | 0.41 | 0.33 | 0.51 | < .001 |
| Education level: low-medium | 0.64 | 0.43 | 0.97 | .033 |
| Education level: medium-high | 0.47 | 0.31 | 0.71 | < .001 |
| Education level: high | 0.42 | 0.26 | 0.65 | < .001 |
| Parental alcohol misuse | 1.71 | 1.25 | 2.35 | .001 |
| Ghanaian |  |  |  |  |
| Emotional neglect | 1.96 | 1.34 | 2.86 | .001 |
| Age | 1.01 | 0.99 | 1.02 | .215 |
| Sex: Female | 0.54 | 0.39 | 0.75 | < .001 |
| Education level: low-medium | 1.27 | 0.85 | 1.90 | .251 |
| Education level: medium-high | 1.22 | 0.78 | 1.92 | .377 |
| Education level: high | 0.53 | 0.22 | 1.28 | .159 |
| Parental alcohol misuse | 2.48 | 1.46 | 4.22 | .001 |
| Turkish |  |  |  |  |
| Emotional neglect | 1.82 | 1.30 | 2.56 | .001 |
| Age | 0.99 | 0.98 | 1.00 | .181 |
| Sex: Female | 0.16 | 0.11 | 0.24 | < .001 |
| Education level: low-medium | 0.85 | 0.53 | 1.34 | .478 |
| Education level: medium-high | 1.06 | 0.67 | 1.69 | .794 |
| Education level: high | 1.20 | 0.72 | 1.98 | .485 |
| Parental alcohol misuse | 3.45 | 2.06 | 5.78 | < .001 |
| Moroccan |  |  |  |  |
| Emotional neglect | 3.38 | 2.24 | 5.10 | < .001 |
| Age | 0.97 | 0.96 | 0.99 | .004 |
| Sex: Female | 0.16 | 0.10 | 0.25 | < .001 |
| Education level: low-medium | 1.64 | 0.84 | 3.22 | .146 |
| Education level: medium-high | 1.28 | 0.66 | 2.46 | .463 |
| Education level: high | 1.90 | 0.96 | 3.73 | .064 |
| Parental alcohol misuse | 3.83 | 1.71 | 8.58 | .001 |

*Note.* AUDIT = alcohol use disorder identification test; CI = confidence interval; LL = lower level; OR = odds ratio; UL = upper level.

Reference group for education level = low.
Pooled results based on multiple imputed datasets.

**Supplementary Table S12b**

Association between psychological abuse and problematic alcohol use (AUDIT ≥ 8 for males, AUDIT ≥ 6 for females) per ethnicity, adjusted for confounding by age, sex, education level, and parental alcohol misuse.

|  | OR | 95% CI for OR | | *P* |
| --- | --- | --- | --- | --- |
|  |  | LL | UL |  |
| Dutch |  |  |  |  |
| Psychological abuse | 1.04 | 0.87 | 1.25 | .641 |
| Age | 0.98 | 0.97 | 0.98 | < .001 |
| Sex: Female | 0.78 | 0.69 | 0.89 | < .001 |
| Education level: low-medium | 0.79 | 0.52 | 1.20 | .276 |
| Education level: medium-high | 1.09 | 0.73 | 1.62 | .664 |
| Education level: high | 1.33 | 0.91 | 1.95 | .142 |
| Parental alcohol misuse | 1.23 | 1.00 | 1.50 | .045 |
| South-Asian Surinamese |  |  |  |  |
| Psychological abuse | 1.59 | 1.19 | 2.13 | .002 |
| Age | 0.97 | 0.96 | 0.98 | < .001 |
| Sex: Female | 0.24 | 0.18 | 0.31 | < .001 |
| Education level: low-medium | 0.77 | 0.52 | 1.12 | .167 |
| Education level: medium-high | 0.53 | 0.35 | 0.81 | .003 |
| Education level: high | 0.68 | 0.45 | 1.03 | .067 |
| Parental alcohol misuse | 2.11 | 1.60 | 2.77 | < .001 |
| African Surinamese |  |  |  |  |
| Psychological abuse | 1.70 | 1.31 | 2.20 | < .001 |
| Age | 0.98 | 0.97 | 0.98 | < .001 |
| Sex: Female | 0.42 | 0.34 | 0.52 | < .001 |
| Education level: low-medium | 0.66 | 0.44 | 0.99 | .045 |
| Education level: medium-high | 0.48 | 0.31 | 0.73 | .001 |
| Education level: high | 0.44 | 0.28 | 0.69 | < .001 |
| Parental alcohol misuse | 1.77 | 1.29 | 2.41 | < .001 |
| Ghanaian |  |  |  |  |
| Psychological abuse | 1.48 | 0.94 | 2.32 | .094 |
| Age | 1.01 | 0.99 | 1.02 | .240 |
| Sex: Female | 0.55 | 0.40 | 0.76 | < .001 |
| Education level: low-medium | 1.26 | 0.84 | 1.89 | .264 |
| Education level: medium-high | 1.23 | 0.79 | 1.93 | .359 |
| Education level: high | 0.53 | 0.22 | 1.29 | .164 |
| Parental alcohol misuse | 2.64 | 1.54 | 4.52 | < .001 |
| Turkish |  |  |  |  |
| Psychological abuse | 1.95 | 1.31 | 2.91 | .001 |
| Age | 0.99 | 0.98 | 1.01 | .221 |
| Sex: Female | 0.17 | 0.11 | 0.24 | < .001 |
| Education level: low-medium | 0.83 | 0.53 | 1.32 | .431 |
| Education level: medium-high | 1.04 | 0.65 | 1.64 | .879 |
| Education level: high | 1.16 | 0.70 | 1.92 | .564 |
| Parental alcohol misuse | 3.46 | 2.07 | 5.81 | < .001 |
| Moroccan |  |  |  |  |
| Psychological abuse | 3.69 | 2.40 | 5.69 | < .001 |
| Age | 0.98 | 0.96 | 0.99 | .011 |
| Sex: Female | 0.16 | 0.10 | 0.25 | < .001 |
| Education level: low-medium | 1.76 | 0.90 | 3.43 | .100 |
| Education level: medium-high | 1.38 | 0.71 | 2.66 | .338 |
| Education level: high | 1.95 | 0.99 | 3.84 | .052 |
| Parental alcohol misuse | 4.21 | 1.93 | 9.18 | < .001 |

*Note.* AUDIT = alcohol use disorder identification test; CI = confidence interval; LL = lower level; OR = odds ratio; UL = upper level.

Reference group for education level = low.
Pooled results based on multiple imputed datasets.

**Supplementary Table S12c**

Association between physical abuse and problematic alcohol use (AUDIT ≥ 8 for males, AUDIT ≥ 6 for females) per ethnicity, adjusted for confounding by age, sex, education level, and parental alcohol misuse.

|  | OR | 95% CI for OR | | *P* |
| --- | --- | --- | --- | --- |
|  |  | LL | UL |  |
| Dutch |  |  |  |  |
| Physical abuse | 1.10 | 0.88 | 1.36 | .409 |
| Age | 0.98 | 0.97 | 0.98 | < .001 |
| Sex: Female | 0.78 | 0.69 | 0.89 | < .001 |
| Education level: low-medium | 0.80 | 0.53 | 1.21 | .284 |
| Education level: medium-high | 1.10 | 0.74 | 1.63 | .652 |
| Education level: high | 1.34 | 0.91 | 1.96 | .136 |
| Parental alcohol misuse | 1.23 | 1.01 | 1.49 | .042 |
| South-Asian Surinamese |  |  |  |  |
| Physical abuse | 1.73 | 1.30 | 2.29 | < .001 |
| Age | 0.97 | 0.96 | 0.98 | < .001 |
| Sex: Female | 0.24 | 0.19 | 0.32 | < .001 |
| Education level: low-medium | 0.77 | 0.53 | 1.13 | .176 |
| Education level: medium-high | 0.53 | 0.35 | 0.80 | .002 |
| Education level: high | 0.67 | 0.45 | 1.02 | .060 |
| Parental alcohol misuse | 2.11 | 1.61 | 2.77 | < .001 |
| African Surinamese |  |  |  |  |
| Physical abuse | 1.58 | 1.24 | 2.02 | < .001 |
| Age | 0.98 | 0.97 | 0.98 | < .001 |
| Sex: Female | 0.43 | 0.34 | 0.53 | < .001 |
| Education level: low-medium | 0.65 | 0.43 | 0.97 | .035 |
| Education level: medium-high | 0.47 | 0.31 | 0.71 | < .001 |
| Education level: high | 0.42 | 0.27 | 0.66 | < .001 |
| Parental alcohol misuse | 1.82 | 1.33 | 2.48 | < .001 |
| Ghanaian |  |  |  |  |
| Physical abuse | 2.18 | 1.53 | 3.10 | < .001 |
| Age | 1.01 | 0.99 | 1.02 | .244 |
| Sex: Female | 0.56 | 0.41 | 0.77 | < .001 |
| Education level: low-medium | 1.25 | 0.83 | 1.87 | .288 |
| Education level: medium-high | 1.19 | 0.76 | 1.87 | .449 |
| Education level: high | 0.50 | 0.20 | 1.21 | .123 |
| Parental alcohol misuse | 2.48 | 1.46 | 4.21 | .001 |
| Turkish |  |  |  |  |
| Physical abuse | 2.38 | 1.64 | 3.46 | < .001 |
| Age | 0.99 | 0.98 | 1.00 | .134 |
| Sex: Female | 0.17 | 0.12 | 0.25 | < .001 |
| Education level: low-medium | 0.81 | 0.51 | 1.28 | .362 |
| Education level: medium-high | 1.01 | 0.63 | 1.60 | .970 |
| Education level: high | 1.08 | 0.65 | 1.80 | .761 |
| Parental alcohol misuse | 3.42 | 2.04 | 5.73 | < .001 |
| Moroccan |  |  |  |  |
| Physical abuse | 5.20 | 3.45 | 7.82 | < .001 |
| Age | 0.97 | 0.96 | 0.99 | .006 |
| Sex: Female | 0.18 | 0.11 | 0.28 | < .001 |
| Education level: low-medium | 1.78 | 0.91 | 3.48 | .093 |
| Education level: medium-high | 1.38 | 0.72 | 2.68 | .334 |
| Education level: high | 1.84 | 0.93 | 3.63 | .079 |
| Parental alcohol misuse | 3.52 | 1.57 | 7.92 | .003 |

*Note.* AUDIT = alcohol use disorder identification test; CI = confidence interval; LL = lower level; OR = odds ratio; UL = upper level.

Reference group for education level = low.
Pooled results based on multiple imputed datasets.

**Supplementary Table S12d**

Association between sexual abuse and problematic alcohol use (AUDIT ≥ 8 for males, AUDIT ≥ 6 for females) per ethnicity, adjusted for confounding by age, sex, education level, and parental alcohol misuse.

|  | OR | 95% CI for OR | | *P* |
| --- | --- | --- | --- | --- |
|  |  | LL | UL |  |
| Dutch |  |  |  |  |
| Sexual abuse | 1.37 | 1.12 | 1.68 | .002 |
| Age | 0.97 | 0.97 | 0.98 | < .001 |
| Sex: Female | 0.75 | 0.66 | 0.86 | < .001 |
| Education level: low-medium | 0.79 | 0.52 | 1.20 | .276 |
| Education level: medium-high | 1.09 | 0.73 | 1.62 | .669 |
| Education level: high | 1.33 | 0.91 | 1.95 | .142 |
| Parental alcohol misuse | 1.21 | 0.99 | 1.47 | .060 |
| South-Asian Surinamese |  |  |  |  |
| Sexual abuse | 2.25 | 1.43 | 3.53 | < .001 |
| Age | 0.97 | 0.96 | 0.98 | < .001 |
| Sex: Female | 0.22 | 0.17 | 0.29 | < .001 |
| Education level: low-medium | 0.76 | 0.52 | 1.12 | .165 |
| Education level: medium-high | 0.53 | 0.35 | 0.80 | .002 |
| Education level: high | 0.67 | 0.44 | 1.01 | .055 |
| Parental alcohol misuse | 2.22 | 1.70 | 2.91 | < .001 |
| African Surinamese |  |  |  |  |
| Sexual abuse | 1.50 | 1.07 | 2.09 | .018 |
| Age | 0.98 | 0.97 | 0.99 | < .001 |
| Sex: Female | 0.40 | 0.32 | 0.51 | < .001 |
| Education level: low-medium | 0.65 | 0.44 | 0.98 | .040 |
| Education level: medium-high | 0.47 | 0.31 | 0.71 | < .001 |
| Education level: high | 0.42 | 0.27 | 0.67 | < .001 |
| Parental alcohol misuse | 1.87 | 1.37 | 2.54 | < .001 |
| Ghanaian |  |  |  |  |
| Sexual abuse | 2.00 | 1.18 | 3.39 | .010 |
| Age | 1.01 | 0.99 | 1.02 | .222 |
| Sex: Female | 0.54 | 0.39 | 0.74 | < .001 |
| Education level: low-medium | 1.24 | 0.82 | 1.85 | .305 |
| Education level: medium-high | 1.19 | 0.76 | 1.87 | .442 |
| Education level: high | 0.49 | 0.20 | 1.21 | .122 |
| Parental alcohol misuse | 2.71 | 1.60 | 4.58 | < .001 |
| Turkish |  |  |  |  |
| Sexual abuse | 4.00 | 2.11 | 7.59 | < .001 |
| Age | 0.99 | 0.98 | 1.01 | .241 |
| Sex: Female | 0.16 | 0.11 | 0.23 | < .001 |
| Education level: low-medium | 0.82 | 0.52 | 1.30 | .396 |
| Education level: medium-high | 1.02 | 0.64 | 1.62 | .944 |
| Education level: high | 1.14 | 0.69 | 1.90 | .605 |
| Parental alcohol misuse | 3.90 | 2.34 | 6.51 | < .001 |
| Moroccan |  |  |  |  |
| Sexual abuse | 5.34 | 3.07 | 9.28 | < .001 |
| Age | 0.97 | 0.96 | 0.99 | .005 |
| Sex: Female | 0.15 | 0.10 | 0.25 | < .001 |
| Education level: low-medium | 1.58 | 0.81 | 3.10 | .181 |
| Education level: medium-high | 1.25 | 0.65 | 2.42 | .501 |
| Education level: high | 1.70 | 0.86 | 3.36 | .124 |
| Parental alcohol misuse | 3.98 | 1.76 | 8.98 | .001 |

*Note.* AUDIT = alcohol use disorder identification test; CI = confidence interval; LL = lower level; OR = odds ratio; UL = upper level.

Reference group for education level = low.
Pooled results based on multiple imputed datasets.

**Supplementary Table S13**

Associations between child maltreatment types and problematic alcohol use (AUDIT ≥ 8 for males, AUDIT ≥ 6 for females) per ethnicity, adjusted for confounding by age, sex, education level, and parental alcohol misuse.

|  | OR | 95% CI for OR | | *P* |
| --- | --- | --- | --- | --- |
|  |  | LL | UL |  |
| Dutch |  |  |  |  |
| Emotional neglect | 1.27 | 1.07 | 1.51 | .006 |
| Psychological abuse | 0.85 | 0.67 | 1.06 | .152 |
| Physical abuse | 1.03 | 0.80 | 1.32 | .837 |
| Sexual abuse | 1.33 | 1.08 | 1.64 | .007 |
| Age | 0.97 | 0.97 | 0.98 | < .001 |
| Sex: Female | 0.75 | 0.66 | 0.85 | < .001 |
| Education level: low-medium | 0.80 | 0.53 | 1.22 | .299 |
| Education level: medium-high | 1.08 | 0.73 | 1.61 | .703 |
| Education level: high | 1.32 | 0.90 | 1.93 | .161 |
| Parental alcohol misuse | 1.16 | 0.94 | 1.42 | .159 |
| South-Asian Surinamese |  |  |  |  |
| Emotional neglect | 1.43 | 0.99 | 2.06 | .058 |
| Psychological abuse | 0.93 | 0.60 | 1.44 | .746 |
| Physical abuse | 1.38 | 0.95 | 2.00 | .090 |
| Sexual abuse | 1.80 | 1.12 | 2.88 | .015 |
| Age | 0.97 | 0.96 | 0.98 | < .001 |
| Sex: Female | 0.22 | 0.17 | 0.29 | < .001 |
| Education level: low-medium | 0.76 | 0.52 | 1.12 | .164 |
| Education level: medium-high | 0.52 | 0.35 | 0.79 | .002 |
| Education level: high | 0.65 | 0.43 | 0.99 | .044 |
| Parental alcohol misuse | 1.97 | 1.49 | 2.61 | < .001 |
| African Surinamese |  |  |  |  |
| Emotional neglect | 1.49 | 1.06 | 2.09 | .021 |
| Psychological abuse | 1.12 | 0.74 | 1.70 | .582 |
| Physical abuse | 1.17 | 0.84 | 1.62 | .362 |
| Sexual abuse | 1.16 | 0.81 | 1.66 | .423 |
| Age | 0.98 | 0.97 | 0.99 | < .001 |
| Sex: Female | 0.40 | 0.32 | 0.51 | < .001 |
| Education level: low-medium | 0.65 | 0.43 | 0.97 | .036 |
| Education level: medium-high | 0.47 | 0.31 | 0.71 | < .001 |
| Education level: high | 0.42 | 0.27 | 0.66 | < .001 |
| Parental alcohol misuse | 1.67 | 1.21 | 2.29 | .002 |
| Ghanaian |  |  |  |  |
| Emotional neglect | 1.73 | 1.05 | 2.84 | .032 |
| Psychological abuse | 0.55 | 0.29 | 1.02 | .059 |
| Physical abuse | 2.07 | 1.32 | 3.23 | .001 |
| Sexual abuse | 1.65 | 0.94 | 2.90 | .082 |
| Age | 1.01 | 0.99 | 1.02 | .213 |
| Sex: Female | 0.54 | 0.39 | 0.74 | < .001 |
| Education level: low-medium | 1.24 | 0.82 | 1.86 | .310 |
| Education level: medium-high | 1.16 | 0.74 | 1.82 | .521 |
| Education level: high | 0.47 | 0.19 | 1.15 | .099 |
| Parental alcohol misuse | 2.40 | 1.40 | 4.12 | .002 |
| Turkish |  |  |  |  |
| Emotional neglect | 1.29 | 0.84 | 1.98 | .242 |
| Psychological abuse | 1.04 | 0.61 | 1.77 | .881 |
| Physical abuse | 1.83 | 1.15 | 2.92 | .011 |
| Sexual abuse | 2.77 | 1.41 | 5.45 | .003 |
| Age | 0.99 | 0.97 | 1.00 | .112 |
| Sex: Female | 0.16 | 0.11 | 0.23 | < .001 |
| Education level: low-medium | 0.80 | 0.50 | 1.27 | .349 |
| Education level: medium-high | 1.00 | 0.63 | 1.60 | .996 |
| Education level: high | 1.05 | 0.63 | 1.76 | .842 |
| Parental alcohol misuse | 3.16 | 1.85 | 5.40 | < .001 |
| Moroccan |  |  |  |  |
| Emotional neglect | 1.33 | 0.74 | 2.40 | .343 |
| Psychological abuse | 1.15 | 0.61 | 2.17 | .663 |
| Physical abuse | 3.43 | 1.98 | 5.96 | < .001 |
| Sexual abuse | 2.57 | 1.39 | 4.75 | .003 |
| Age | 0.97 | 0.95 | 0.99 | .003 |
| Sex: Female | 0.16 | 0.10 | 0.26 | < .001 |
| Education level: low-medium | 1.68 | 0.85 | 3.30 | .134 |
| Education level: medium-high | 1.35 | 0.70 | 2.61 | .371 |
| Education level: high | 1.72 | 0.87 | 3.40 | .118 |
| Parental alcohol misuse | 2.94 | 1.28 | 6.75 | .012 |

*Note.* AUDIT = alcohol use disorder identification test; CI = confidence interval; LL = lower level; OR = odds ratio; UL = upper level.

For each ethnicity, all types of child maltreatment were included in the same model.

Reference group for education level = low.
Pooled results based on multiple imputed datasets.
